# ReTimeML: a retention time predictor that supports the LC–MS/MS analysis of sphingolipids

**DOI:** 10.1038/s41598-024-53860-0

**Published:** 2024-02-22

**Authors:** Michael Allwright, Boris Guennewig, Anna E. Hoffmann, Cathrin Rohleder, Beverly Jieu, Long H. Chung, Yingxin C. Jiang, Bruno F. Lemos Wimmer, Yanfei Qi, Anthony S. Don, F. Markus Leweke, Timothy A. Couttas

**Affiliations:** 1 ForeFront, Brain and Mind Centre, The University of Sydney, Sydney, Australia; 2 Translational Research Collective, Brain and Mind Centre, The University of Sydney, Sydney, NSW 2006 Australia; 3 Endosane Pharmaceuticals GmbH, Berlin, Germany; 4 Department of Psychiatry and Psychotherapy, Central Institute of Mental Health, Medical Faculty Mannheim, Heidelberg University, Mannheim, Germany; 5 Centenary Institute, The University of Sydney, Sydney, Australia; 6 School of Medical Sciences, Faculty of Medicine and Health, The University of Sydney, Sydney, Australia

**Keywords:** Ceramide, Sphingomyelin, LC–MS/MS, Retention time, Regression modelling, Lasso, Ridge, Python, Streamlit, Serum, Cerebrospinal fluid, Sphingolipids, Mass spectrometry

## Abstract

The analysis of ceramide (Cer) and sphingomyelin (SM) lipid species using liquid chromatography–tandem mass spectrometry (LC–MS/MS) continues to present challenges as their precursor mass and fragmentation can correspond to multiple molecular arrangements. To address this constraint, we developed ReTimeML, a freeware that automates the expected retention times (RTs) for Cer and SM lipid profiles from complex chromatograms. ReTimeML works on the principle that LC–MS/MS experiments have pre-determined RTs from internal standards, calibrators or quality controls used throughout the analysis. Employed as reference RTs, ReTimeML subsequently extrapolates the RTs of unknowns using its machine-learned regression library of mass-to-charge (*m/z*) versus RT profiles, which does not require model retraining for adaptability on different LC–MS/MS pipelines. We validated ReTimeML RT estimations for various Cer and SM structures across different biologicals, tissues and LC–MS/MS setups, exhibiting a mean variance between 0.23 and 2.43% compared to user annotations. ReTimeML also aided the disambiguation of SM identities from isobar distributions in paired serum-cerebrospinal fluid from healthy volunteers, allowing us to identify a series of non-canonical SMs associated between the two biofluids comprised of a polyunsaturated structure that confers increased stability against catabolic clearance.

## Introduction

Sphingolipids (SLs) are a diverse family of structural and signalling lipids that comprise a broad range of biological functions crucial to normal physiology, cell signalling and trophic support^1–3^. SLs are defined by their sphingoid backbone, which in mammals consists predominantly of an 1, 3-dihydroxy, 18-carbon, mono-unsaturated sphingosine (d18:1), with variations to this long chain base also studied, including saturated dihydrosphingosine or sphinganine (d18:0), the di-unsaturated sphingadiene (d18:2), as well as mono- (m18:X) and trihydroxy (t18:X) configurations^2,4^.

**Figure 1:**
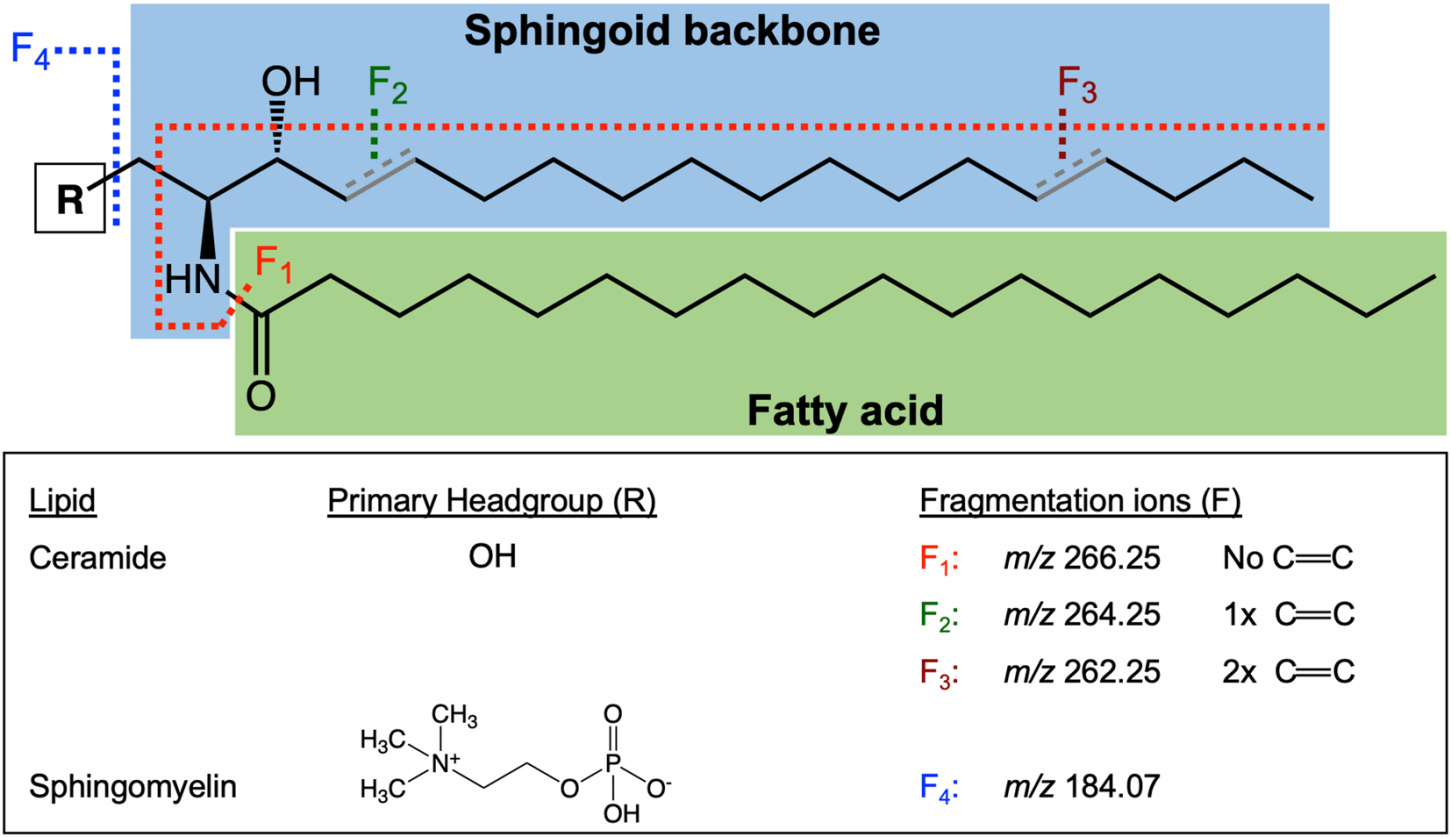
Basic chemical structure of sphingolipids, displaying the primary headgroups (‘R’) for ceramide and sphingomyelin, as well as the sites of characteristics fragmentation ions (‘F’) on the sphingoid base (F_1-3_) and choline headgroup (F_4_) specific to sphingomyelin. Common sites of unsaturation (C=C) on the mammalian sphingoid backbone are also illustrated, and their impact on the reported *m/z* fragment ions.

Ceramide (Cer) is the central intermediate of the sphingolipid pathway, that consists of a sphingoid base amide-linked to a fatty acid of variable length, hydroxylation and degree of unsaturation^5,6^. Modifications to the C1 hydroxyl group of Cer allow for the formation of the more complex glycosylated SLs (e.g., cerebrosides, gangliosides), and the phosphorylated sphingomyelins (SM), which are the most abundant SL class in the plasma membrane of eukaryotes^7^. SM is frequently studied alongside Cer, owing to the functional importance of Cer-SM balance in cellular and inflammatory processes, as well as in ordered domain function (e.g., lipid rafts)^8–11^. Liquid chromatography-tandem mass spectrometry (LC–MS/MS) is the conventional approach for the analysis of Cer and SM lipids, as their fragmentation following positive ionisation ([M + H]^+^) generates characteristic ions of their sphingoid backbone (e.g., *m/z* 264 for d18:1) and side groups (e.g., *m/z* 184 for the choline headgroup of SM) to facilitate their identification (Fig. 1). Their fragmentation can also elucidate structural features of the fatty acyl and sphingoid base chain, including length and degree(s) of unsaturation (Fig. 1), which can help determine the precise position of carbon–carbon double bonds with specialised instruments^4,12^.

Specific precursor–product ion transitions can be employed in selective (SRM) or multiple (MRM) reaction monitoring experiments to improve the accuracy and reproducibility of SL analysis^13–15^, and are readily adapted into commercial (e.g., LipidSearch, LipidBlast, LipidAnnotator) and freeware toolboxes for the development of automated workflows (including setup, data acquisition and validation), thereby making their analysis amendable to non-experts^16–18^. However, resolving SL identities remains complex, as variations in headgroups, carbon backbone length and sites of unsaturation increase the probable number of variants with similar transitions, including isobaric and isomeric species^19^, that cannot be distinguished even with high-resolution instruments^20^. In addition, new structural species are continually being identified^21,22^, adding to this growing complexity.

**Figure 2:**
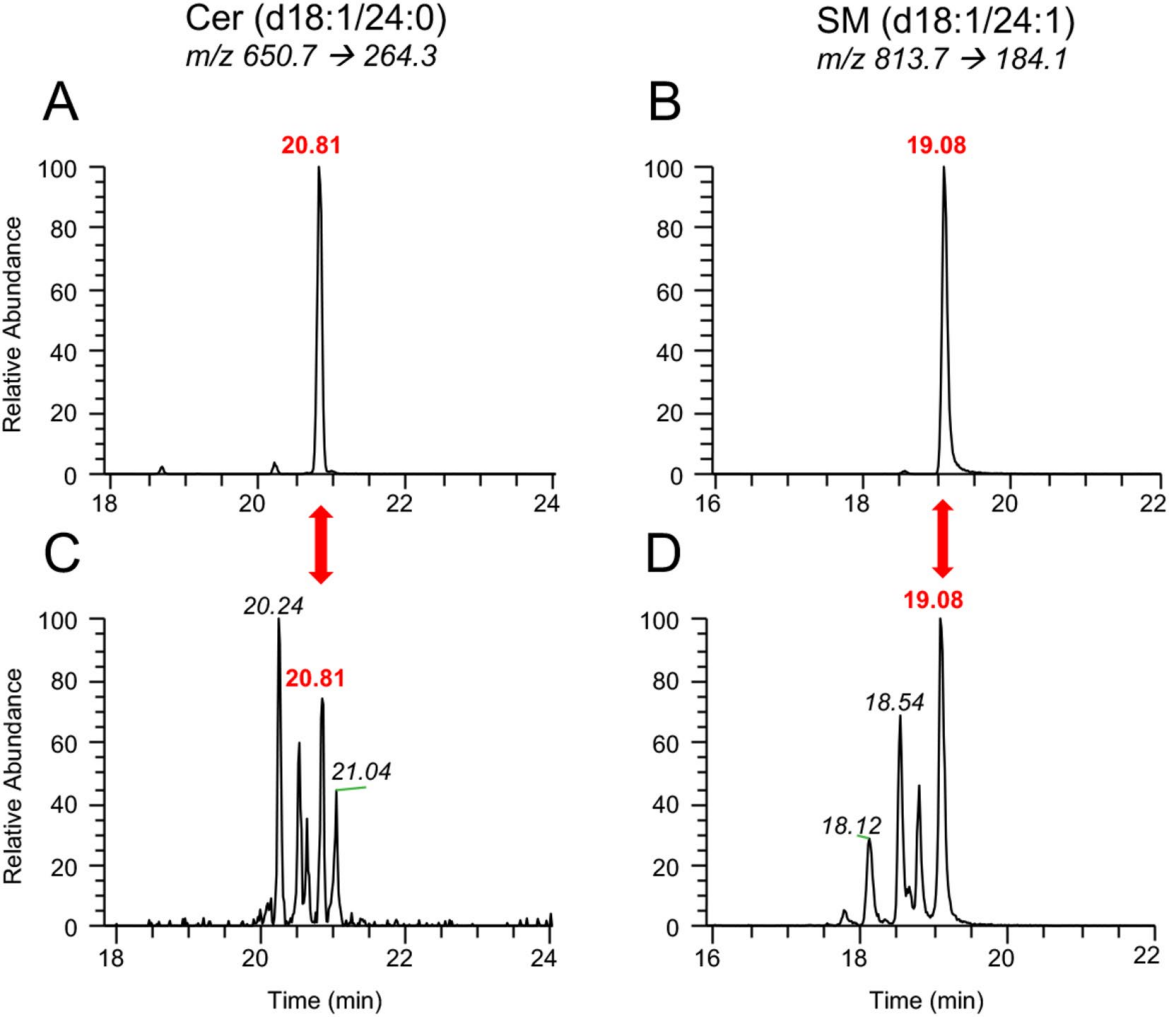
Representative total ion chromatograms for ceramide and sphingomyelin species from (**A**,**B**) pure compounds and (**C**,**D**) biological extracts. Loaded calibrators for (**A**) Cer(d18:1/24:0) and (**B**) SM(d18:1/24:1) allow us to resolve their position in the samples (**C**,**D**), which have multiple peaks assigned to their MRM scan, caused by interfering ions with similar precursor-product transitions.

Notwithstanding, the resolution of closely related SLs, including those with similar structures or nominal mass, can be achieved through LC separation, augmenting the accuracy of MS/MS assignments^23–25^. SL identities can be further validated by matching their chromatographic separation or retention time (RT) in complex samples against pure external standards or internal (deuterated) compounds (Fig. 2)^26^. Though this unequivocally resolves their identities, purchasing standards for all SLs of interest is not feasible, given many are not commercially available and the financial burden of purchasing copious numbers of SL variants.

Advancements in computational predictions have vastly improved RT estimation across various lipids (including SLs) and small molecules, for both targeted (SRM/MRM) and global lipid (untargeted) analyses^27–34^. However, numerous selective modifications to lipid separation (e.g., sample preparation, appropriate solvents, flow rate, chromatography matrix) are made to improve their detection and avoid batch effects. This can impose limitations on consistent RT estimations as modelling is often confined to comparable experimental conditions, including tissue type, potential class-specific biases and/or requires frequent retraining and validation^30,33,34^. This has precluded the capacity for robust or straightforward integration of RT estimates in lipid identification tools and experimental analyses.

**Figure 3:**
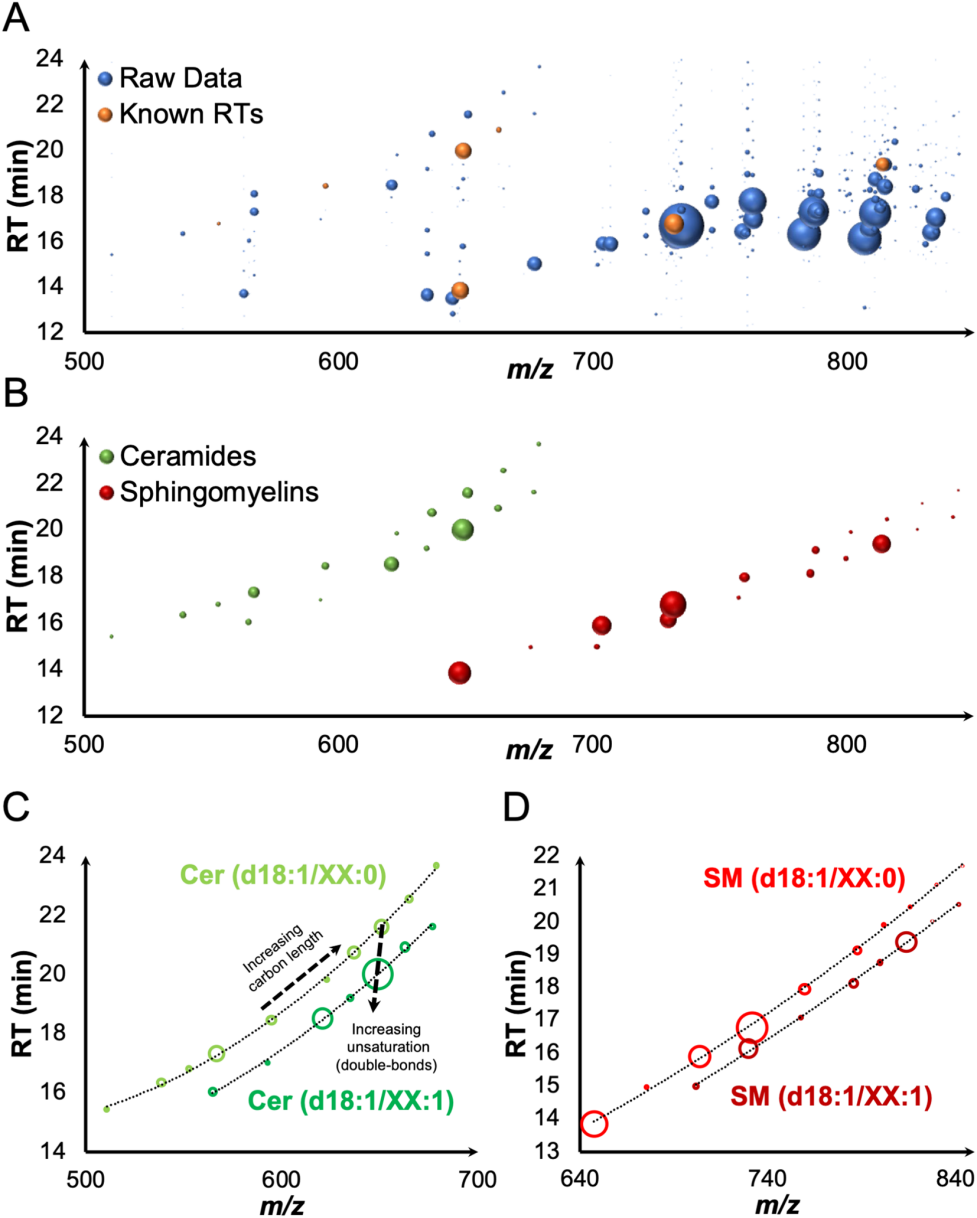
2D plots of MRET profiles. (**A**) Each bubble represents a distinct ion and its relative TIC peak area (bubble size) identified during an SRM/MRM experiment, with the identity of ions for Cer and SM standards/internal controls highlighted. These values were assigned as points of reference to (**B**) extrapolate the elution profiles of Cer and SM, which subsequently allows for the removal of interfering ions. Enhanced views of elution profiles for (**C**) Cer and (**D**) SM demonstrate how relative RT can assist with determining structural characteristics, in this instance relative RT helps identify effects on SLs from increasing carbon chain length and degree of unsaturation (C=C bonds). 2D plots were derived from data obtained in our previous study^14^.

To overcome these limitations in the assessment of SLs, Mass versus Relative Elution Time (MRET) profiling can be employed^35^, plotting two-dimensional data of the SLs nominated precursor mass (*m/z*), against their elution time (RT) (Fig. 3). Standards and controls (quality or internal) are utilised as “points of reference”, with the generated 2D plots used to visually extrapolate the position of other SLs within that class, using the knowledge of their known mass and characteristic fragmentation ions (i.e,. sphingoid backbone, C1 head group) as molecular descriptors to determine the RT of unknowns (Fig. 3). Recognising MRET patterns specific to a given SL family (Fig. 3B), as well as structural characteristics (e.g., degree of unsaturation, Fig. 3C,D), allows for the elimination of major interferences, resulting from ions of indistinguishable precursor and product fragmentation.

Presently, MRET profiling is manually performed. In this study, we aimed to automate the process by developing a bespoke, web-based tool entitled ReTimeML, aptly named to describe the task at hand—a calculated Remeasurement of the Time, on column, to predict the elution of (sphingo)lipids. ReTimeML was constructed through Machine-Learned regression of user RT assignments, collected from our database of LC–MS/MS analyses on Cer and SM lipids, and literature sources. Herein, we verified ReTimeML’s capacity to accurately annotate (> 99% accuracy) Cer and SM RTs, compared to expert-user assignments, across multiple tissues and LC–MS/MS experimental conditions. Notably, ReTimeML was successfully applied to aid the identification of noncanonical Cer and SM structures, resolve ion interferences, and guide the accurate annotations for Cer and SM expressional differences in cerebrospinal fluid (CSF) and paired serum collected from the same healthy volunteers (HVs).

## Results

### Regression model development and selection

**Figure 4:**
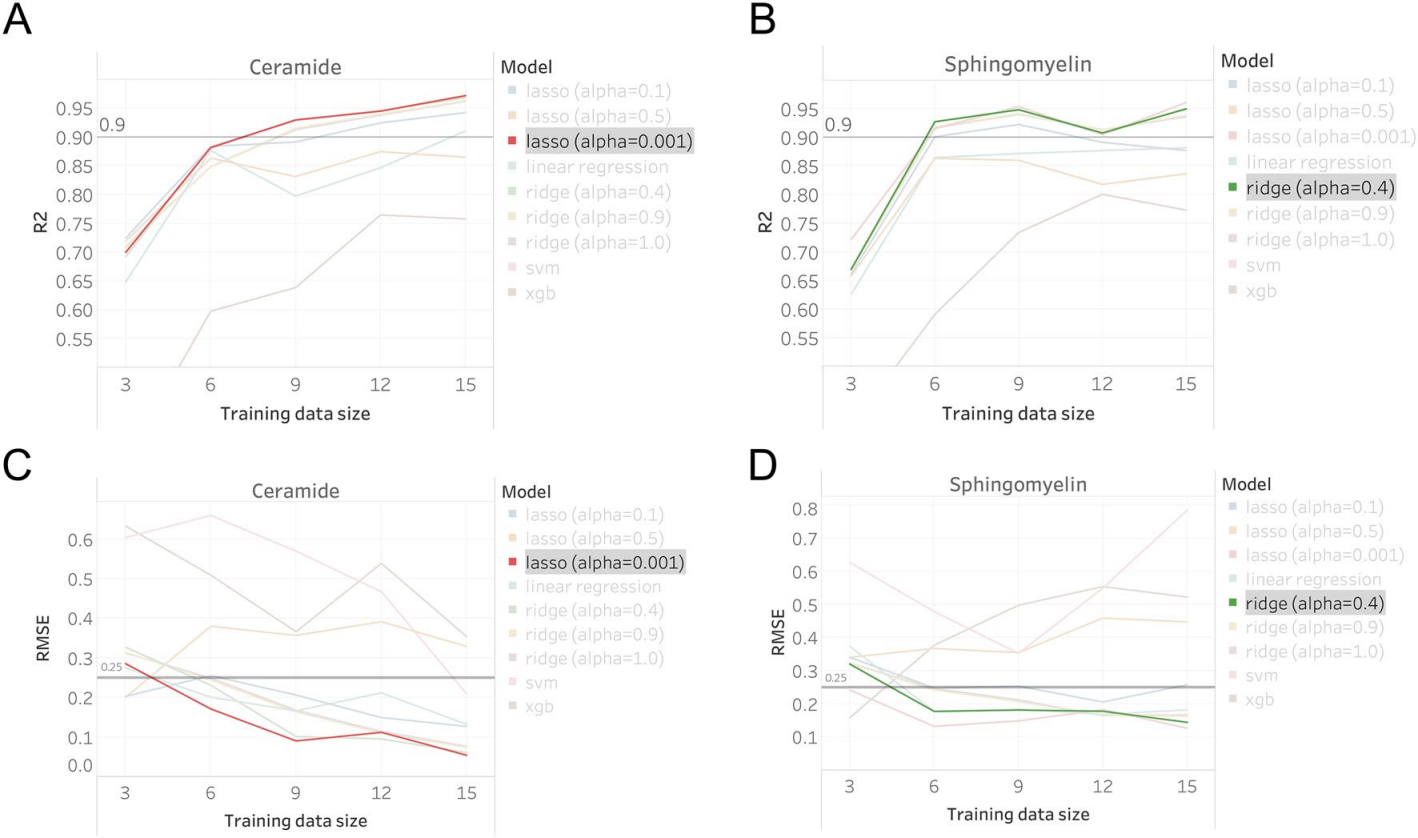
The (**A**,**B**) coefficient of determination (R^2^) and (**C**,**D**) root-mean-square-error (RMSE) values for every regression model applied to the validation data with increasing training sample size. The selected regression model for (**A**,**C**) ceramide (lasso, alpha = 0.001, red line) and (**B**,**D**) sphingomyelin (ridge, alpha = 0.4, green line) has been emphasised.

We assessed the capacity of the nominated regression algorithms (Supplementary Data 1) to learn from descriptor information for identifying Cer and SM lipids (e.g., precursor mass, fragmentation) and user RT annotations of previous work (Supplementary Data 2). RT data was sequentially increased to determine the optimal training sample size, broken down by molecular features, for which regression model performance (evaluated on validation data) yielded coefficient of determination (R^2^) and root mean squared error (RMSE) values at the acceptance thresholds (R^2^ > 0.9, RMSE < 0.25), with the fulfilment of both criteria imperative. As anticipated, performance increased across all models when augmenting the training set size (Fig. 4). Lasso (alpha = 0.001) and ridge regression (alpha = 0.4) outperformed the other machine-learned models (Fig. 4), and were assigned as the optimal regression algorithms for Cer and SM RT estimations, respectively. Though comparable, lasso achieved a slightly more favourable R^2^ value for Cer (lasso: 0.930; ridge: 0.929, n = 9), while ridge regression appeared to be more beneficial for SM estimations (lasso: 0.915; ridge: 0.928, n = 6). Lasso yielded marginally lower RMSE values for both Cer (lasso: 0.091; ridge: 0.102, n = 9) and SM (lasso: 0.132; ridge: 0.178, n = 6), when applying the same number of datasets to concurrently meet our R^2^ > 0.9 prerequisites, though both were well-below the acceptable RMSE < 0.25 threshold (Fig. 4, Supplementary Data 2).

### Performance evaluation and model robustness

**Figure 5:**
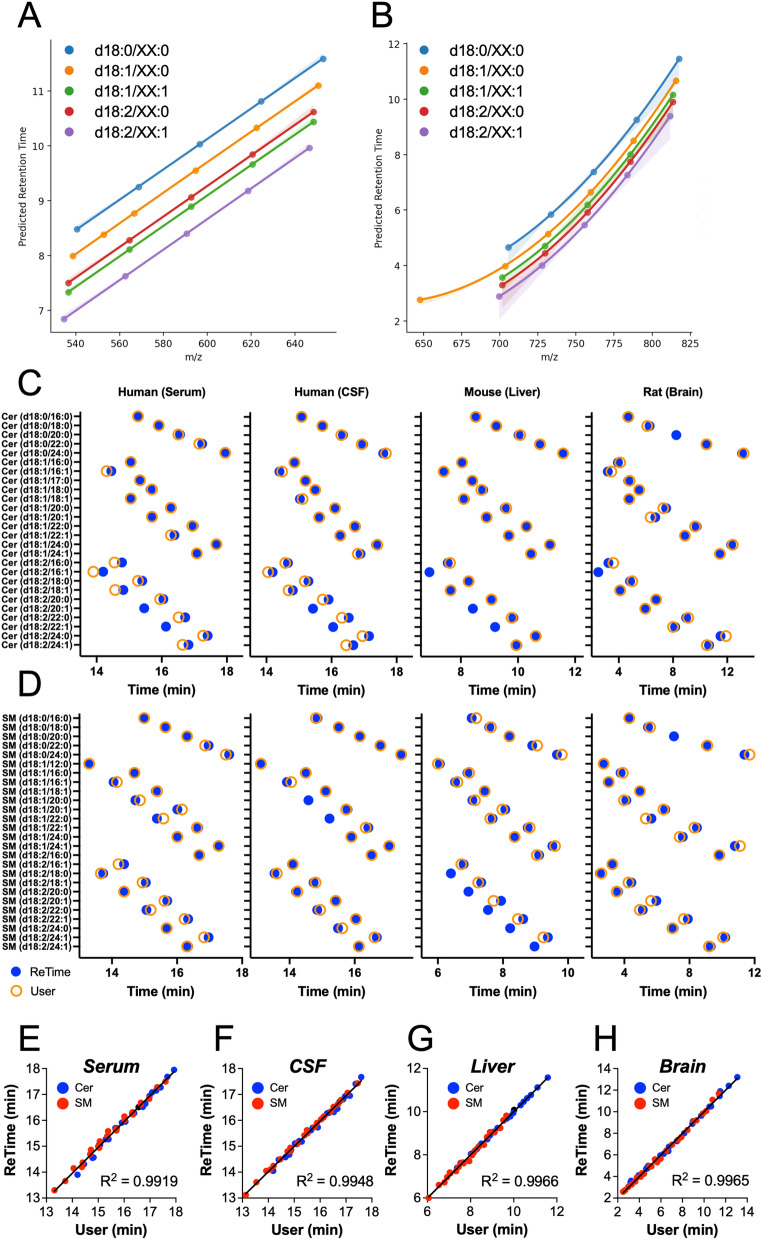
Representative ReTimeML outputs estimating RTs for (**A**) gradient (human serum, ceramide) and (**B**) isocratic (rat brain, sphingomyelin) LC–MS/MS analyses. (**C**,**D**) ReTimeML estimations (blue circle) aligned with user RT annotations (orange circle) for (**C**) Cer and (**D**) SM across our four experimental fluid/tissue analyses. Scatter plots representing ReTimeML estimations vs user-determined RT values for (**E**) human serum (**F**) CSF, (**G**) mouse liver and (**H**) rat brain SL extracts. Squared correlation coefficient (R^2^) are reported. The complete list of RT values (ReTimeML and user) have been provided in Tables 1 and 2.

The performance of ReTimeML’s lasso and ridge regression modelling was verified in four, independently performed, LC–MS/MS analyses on frequently observed d18:0, d18:1 and d18:2 Cer and SM lipid species (Tables 1 and 2). LC–MS/MS for Cer and SM species were performed on various tissues/fluids of rodents and humans, and different chromatography conditions (including an isocratic vs. gradient comparison). Cer and SM species for which experimental RTs were known (i.e., calibrators, internal/quality controls, experimentally determined), were assigned as points of reference (‘Train’), with the remaining unknown RTs extrapolated for each experiment (‘Test’). ReTimeML’s output provides users with an RT list for all Cer/SMs of interest, including those listed as references, which can be downloaded as a .csv file or directly copied into Excel or a similar spreadsheet. An MRET profile plot is also generated that displays the position of each calculated SL, organised into different degrees of SL structural unsaturation. Representative figures of ReTimeML’s output are illustrated in Fig. 5A and B. MRET plots were also manually constructed from user-validated assignments (Supplementary Fig. 1), with their eluting order supporting ReTimeML’s output, as well as previous literature on the separation of these SLs under reverse-phase conditions^35–37^. ReTimeML’s estimations displayed exceptional agreement when compared to user-determined RTs (Fig. 5C,D). ReTimeML predicted the RTs of 192 Cer and SM species, across the four LC–MS/MS experiments, with an average and median prediction error of 7.6 and 3.6 s, respectively, with each validation experiment achieving R^2^ > 0.99 when comparing ReTimeML estimations to experimentally determined RTs (Fig. 5E–H). Of the ReTimeML predicted RTs, 14 deviated more than 3% from user assignments, with errors of this magnitude occurring only when RTs were estimated under isocratic conditions (Tables 1 and 2). For gradient RT estimations, 23 of 142 deviated more than 1%, with only 2 of these estimates exceeding a 2% variance from user assignments (Tables 1 and 2).

**Table 1:** Precursor-product ions used for the analysis of ceramides and comparison between ReTimeML estimations to user-defined RTs.

Sphingolipid	Precursor (*m/z*)	Product (*m/z*)	Serum (human)			CSF (human)			Liver (mouse)			Brain (rat)		
			ReTimeML	User defined*	% error	ReTimeML	User defined*	% error	ReTimeML	User defined*	% error	ReTimeML	User defined*	% error
Cer (d18:2/16:1)	534.5	262.3	14.20	13.9	2.16%	14.19	14.05	1.00%	6.9	ND	–	2.49	ND	–
Cer (d18:2/16:0)	536.5	262.3	14.78	14.55	1.58%	14.66	14.58	0.55%	7.54	7.62	1.05%	3.24	3.6	10.00%
Cer (d18:1/16:1)	536.5	264.3	14.45	14.31	0.98%	14.40	14.48	0.55%	7.38	7.4	0.27%	3.19	3.45	7.54%
Cer (d18:1/16:0)	538.6	264.3	15.03	**15.05**	0.13%	14.88	**14.86**	0.13%	8.02	**8.04**	0.25%	3.94	**4.1**	3.90%
Cer (d18:0/16:0)	540.5	266.3	15.29	**15.27**	0.13%	15.10	**15.08**	0.13%	8.5	**8.52**	0.23%	4.64	**4.7**	1.28%
Cer (d18:1/17:0)	552.7	264.3	15.34	**15.33**	0.07%	15.19	**15.20**	0.07%	8.4	**8.40**	0.00%	4.7	**4.8**	2.08%
Cer (d18:2/18:1)	562.5	262.3	14.82	14.57	1.72%	14.81	14.68	0.89%	7.66	7.64	0.26%	4.06	4.1	0.98%
Cer (d18:2/18:0)	564.5	262.3	15.40	15.27	0.85%	15.29	15.18	0.72%	8.3	8.27	0.36%	4.83	4.99	3.21%
Cer (d18:1/18:1)	564.5	264.3	15.07	**15.05**	0.13%	15.03	**15.11**	0.53%	8.14	**8.10**	0.49%	4.78	**4.77**	0.21%
Cer (d18:1/18:0)	566.6	264.3	15.66	**15.70**	0.25%	15.51	**15.50**	0.06%	8.78	**8.72**	0.69%	5.55	**5.5**	0.91%
Cer (d18:0/18:0)	568.6	266.3	15.91	**15.91**	0.00%	15.72	**15.72**	0.00%	9.25	**9.24**	0.11%	6.27	**6.1**	2.79%
Cer (d18:2/20:1)	590.6	262.3	15.46	NA	–	15.43	NA	–	8.42	ND	–	5.95	5.95	0.00%
Cer (d18:2/20:0)	592.6	262.3	16.05	15.95	0.63%	15.91	15.73	1.14%	9.07	9.07	0.00%	6.74	6.75	0.15%
Cer (d18:1/20:1)	592.6	264.3	15.72	15.7	0.13%	15.65	15.62	0.19%	8.9	8.9	0.00%	6.69	6.35	5.35%
Cer (d18:1/20:0)	594.6	264.3	16.31	**16.28**	0.18%	16.13	**16.11**	0.12%	9.54	**9.59**	0.52%	7.49	**7.28**	2.88%
Cer (d18:0/20:0)	596.6	266.3	16.56	16.51	0.30%	16.35	16.3	0.31%	10.02	10.08	0.60%	8.24	ND	–
Cer (d18:2/22:1)	618.6	262.3	16.13	NA	–	16.05	NA	–	9.2	ND	–	8.16	8	2.00%
Cer (d18:2/22:0)	620.6	262.3	16.72	16.52	1.21%	16.53	16.32	1.29%	9.84	9.78	0.61%	8.97	9.12	1.64%
Cer (d18:1/22:1)	620.6	264.3	16.39	16.28	0.68%	16.27	16.27	0.00%	9.68	9.68	0.00%	8.92	8.88	0.45%
Cer (d18:1/22:0)	622.6	264.3	16.98	**16.94**	0.24%	16.75	**16.72**	0.18%	10.32	**10.3**	0.19%	9.74	**9.63**	1.14%
Cer (d18:0/22:0)	624.6	266.3	17.24	17.15	0.52%	16.97	16.93	0.24%	10.8	10.77	0.28%	10.51	10.47	0.38%
Cer (d18:2/24:1)	646.6	262.3	16.82	16.64	1.08%	16.67	16.45	1.34%	9.98	9.94	0.40%	10.66	10.5	1.52%
Cer (d18:2/24:0)	648.6	262.3	17.41	17.27	0.81%	17.15	16.95	1.18%	10.62	10.62	0.00%	11.5	11.9	3.36%
Cer (d18:1/24:1)	648.6	264.3	17.08	**17.08**	0.00%	16.89	**16.81**	0.48%	10.46	**10.46**	0.00%	11.45	**11.46**	0.09%
Cer (d18:1/24:0)	650.7	264.3	17.67	**17.68**	0.06%	17.37	**17.41**	0.23%	11.1	**11.12**	0.18%	12.29	**12.4**	0.89%
Cer (d18:0/24:0)	652.9	266.3	17.93	**17.95**	0.11%	17.59	**17.67**	0.45%	11.58	**11.58**	0.00%	13.09	**13.2**	0.83%
Average variance between user and predicted RTs					**0.58%**			**0.49%**			**0.28%**			**2.23%**

*NA* not analysed in this experiment, *ND* not detected, as peak assignment could not be reliably differentiated from background in the TIC (S/N < 3).

*Bold denotes RTs of standards (including internal controls) that were assigned as points of reference, with MRET principles used to determine the remaining RTs.

**Table 2:** Precursor–product ions used for the analysis of sphingomyelins and comparison between ReTimeML estimations to user-defined RTs.

Sphingolipid	Precursor (*m/z*)	Product primary (*m/z*)	Product secondary (*m/z*)	Serum (human)			CSF (human)			Liver (mouse)			Brain (rat)		
				ReTimeML	User defined*	% error (%)	ReTimeML	User defined*	% error	ReTimeML	User defined*	% error	ReTimeML	User defined*	% error
SM (d18:1/12:0)	647.5	184	264.3	13.31	**13.3**	0.08	13.13	**13.12**	0.08%	6.06	**6.00**	1.00%	2.69	**2.73**	1.47%
SM (d18:2/16:1)	699.6	184	262.3	13.73	13.66	0.51	13.54	13.61	0.51%	6.40	ND	–	2.50	2.58	3.10%
SM (d18:2/16:0)	701.6	184	262.3	14.37	14.20	1.20	14.08	14.1	0.14%	6.79	6.72	1.04%	3.29	3.26	0.92%
SM (d18:1/16:1)	701.6	184	264.3	14.05	**14.15**	0.71	13.92	**14.05**	0.93%	6.53	**6.60**	1.06%	2.98	**3.04**	1.97%
SM (d18:1/16:0)	703.5	184	264.3	14.69	**14.69**	0.00	14.47	**14.5**	0.21%	6.91	**6.96**	0.72%	3.77	**3.9**	3.33%
SM (d18:0/16:0)	705.6	184	266.3	15.00	14.99	0.07	14.86	14.81	0.34%	7.04	7.18	1.95%	4.25	4.3	1.16%
SM (d18:2/18:1)	727.6	184	262.3	14.40	14.37	0.21	14.20	14.24	0.28%	6.94	ND	–	3.62	3.53	2.55%
SM (d18:2/18:0)	729.6	184	262.3	15.04	14.94	0.67	14.74	14.80	0.41%	7.33	7.24	1.24%	4.43	4.25	4.24%
SM (d18:1/18:1)	729.6	184	264.3	14.72	14.86	0.94	14.58	ND	–	7.06	7.14	1.12%	4.12	3.98	3.52%
SM (d18:1/18:0)	731.6	184	264.3	15.35	**15.39**	0.26	15.13	**15.11**	0.13%	7.45	**7.44**	0.13%	4.94	**4.96**	0.40%
SM (d18:0/18:0)	733.6	184	266.3	15.67	**15.64**	0.19	15.51	**15.51**	0.00%	7.58	**7.63**	0.66%	5.45	**5.58**	2.33%
SM (d18:2/20:1)	755.6	184	262.2	15.06	15.19	0.86	14.85	14.94	0.60%	7.54	ND	–	5.13	4.95	3.64%
SM (d18:2/20:0)	757.6	184	262.3	15.70	15.61	0.58	15.39	15.42	0.19%	7.94	7.71	2.98%	5.97	5.64	5.85%
SM (d18:1/20:1)	757.6	184	264.3	15.38	15.58	1.28	15.23	ND	–	7.68	7.61	0.92%	5.66	5.32	6.39%
SM (d18:1/20:0)	759.6	184	264.3	16.01	16.15	0.87	15.78	15.74	0.25%	8.07	7.98	1.13%	6.51	6.4	1.72%
SM (d18:0/20:0)	761.6	184	266.3	16.32	16.31	0.06	16.16	16.16	0.00%	8.21	8.20	0.12%	7.05	ND	–
SM (d18:2/22:1)	783.7	184	262.3	15.71	15.68	0.19	15.49	15.62	0.83%	8.22	ND	–	7.03	6.95	1.15%
SM (d18:2/22:0)	785.7	184	262.3	16.34	16.23	0.68	16.04	16.04	0.00%	8.62	8.46	1.89%	7.90	7.65	3.27%
SM (d18:1/22:1)	785.7	184	264.3	16.02	16.01	0.06	15.88	15.90	0.13%	8.36	8.36	0.00%	7.59	7.40	2.57%
SM (d18:1/22:0)	787.7	184	264.3	16.65	16.61	0.24	16.42	16.34	0.49%	8.76	8.82	0.68%	8.46	8.30	1.93%
SM (d18:0/22:0)	789.7	184	266.3	16.97	16.86	0.65	16.81	16.79	0.12%	8.90	9.05	1.66%	9.03	9.10	0.77%
SM (d18:2/24:1)	811.7	184	262.3	16.34	16.31	0.18	16.13	16.13	0.00%	8.97	ND	–	9.30	9.20	1.09%
SM (d18:2/24:0)	813.7	184	262.3	16.97	16.83	0.83	16.68	16.61	0.42%	9.38	9.24	1.52%	10.20	10.07	1.29%
SM (d18:1/24:1)	813.7	184	264.3	16.65	**16.68**	0.18	16.52	**16.52**	0.00%	9.11	**9.04**	0.77%	9.89	**9.83**	0.61%
SM (d18:1/24:0)	815.7	184	264.3	17.28	**17.28**	0.00	17.06	**17.06**	0.00%	9.52	**9.60**	0.83%	10.79	**11.10**	2.79%
SM (d18:0/24:0)	817.7	184	266.3	17.60	17.50	0.57	17.45	17.43	0.11%	9.66	9.81	1.53%	11.38	11.69	2.65%
Average variance between user and predicted RTs			0.46%			0.26			1.09%			2.43%			

*NA* not analysed in MRM experiment, *ND* not detected, as peak assignment could not be reliably differentiated from background in the TIC (S/N < 3).

*Bold denotes RTs that were assigned as points of reference with MRET principles used to determine the remaining RTs.

### Accuracy threshold assessment

ReTimeML’s performance in our verification studies prompted us to evaluate how its modelling accuracy responds to variations in the number and/or type (i.e., structure) of reference material employed. This was performed to ascertain the minimum requirement of reference material (‘Train’) that will achieve, on average, appropriate levels of accuracy (< 3% deviation from experimentally determined values). To investigate this potential constraint, we undertook a random sampling of our user-defined RTs from the verification LC–MS/MS experiments (Tables 1 and 2), which were then utilised as operational ‘Train’ values to evaluate their impact on ReTimeML’s subsequent extrapolation of unknowns. Nominated values were incrementally increased to assess the effects of fatty acyl chain length within a sphingoid base (e.g., d18:1/16:0 → d18:1/24:0), alongside structural variations from degree(s) of unsaturation (d18:0/XX:0, d18:1/XX:0, d18:1/XX:1, d18:2/XX:0 and d18:2/XX:1). In all random samplings, the internal control was maintained, with ReTimeML requiring a minimum of two points of reference to begin the estimation of unknowns.

**Figure 6:**
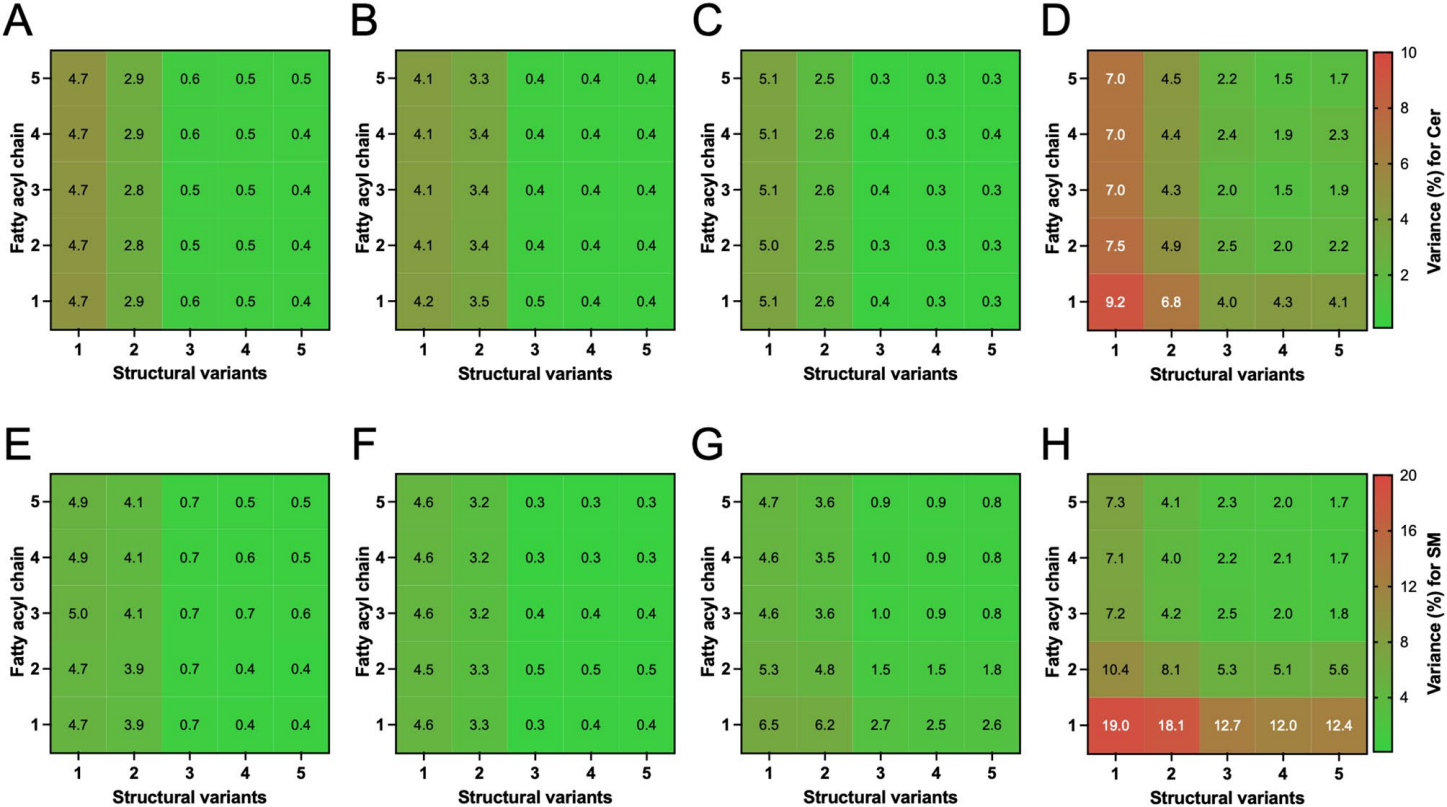
Heat maps of comparative assessment in the accuracy of ReTimeML estimations against the selection and number of reference RTs employed. A random sampling of predetermined RTs was performed within each of our validation cohorts, which incrementally increased the number of references based on fatty-acyl chain length (y-axis) and sphingoid base unsaturation (x-axis). Variance (% error) in ReTimeML estimations for (**A**–**D**) Cer and (**E**–**H**) SM from user-defined RTs are represented for (**A**,**E**) human, serum; (**B**,**F**) human, CSF; (**C**,**G**) mouse, liver and (**D**,**H**), rat, brain. Accuracy was arbitrarily deemed appropriate if ReTimeML variance from user-RTs was less than 3%.

Regardless of the selected RT material used, ReTimeML’s estimations on gradient LC–MS/MS experiments consistently outperformed isocratic measures, requiring fewer ‘Train’ points to achieve appropriate levels of accuracy (Fig. 6). ReTimeML estimations for gradient assessments were deemed suitable upon the employment of three structural variants (e.g., d18:0/XX:0, d18:1/XX:0 and d18:1/XX:1) as reference material, with average deviations from user-RTs ranging from 0.39 to 0.58% for Cer and 0.3–2.71% for SM (Fig. 6A–C,E–G). Increasing the number of reference points beyond this value does not greatly improve RT accuracy, with the exception of SMs assessed in mouse liver tissue where additional fatty acyl chains of the three structural variants improved accuracy to 1% and below (Fig. 6G). For isocratic LC–MS/MS, a larger number of references (n = 5–7) were required to achieve appropriate levels of accuracy (< 3%), with ReTimeML unable to attain a variance from user-RTs below ~ 1.5% (Fig. 6D,H). These results mirrored the similar numbers of ‘Train’ values and model accuracy achieved in our verification experiments (Tables 1 and 2). In all assessments, increasing the number of fatty acyl chain ‘Train’ points does not as drastically improve RT accuracy when compared to increasing the number of structural variants.

### ReTimeML SL resolution in the absence of fragmentation distinguishment.

**Figure 7:**
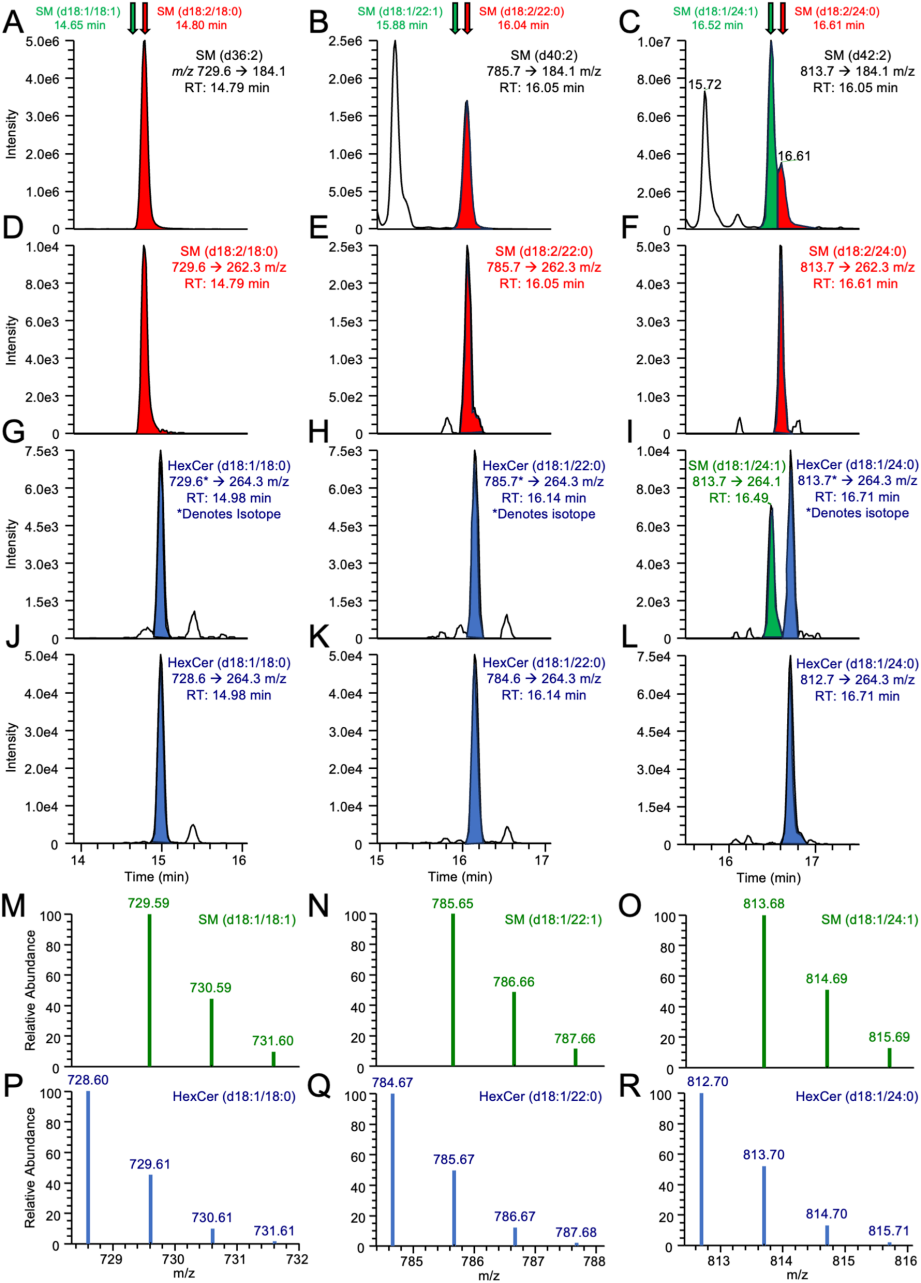
ReTimeML assigned RTs help to resolve disambiguation from isobaric and isotopic interferences in MRM analysis of SMs. The TICs for (**A**–**C**) MRM transitions *m/z* 729.6, 785.7 and 813.7 → 184.1 correspond to SM (d36:2), SM (d40:2), SM (d42:2), respectively. ReTimeML estimated RTs for the SM (d18:1/XX:1) (green arrow/peak) and SM (d18:2/XX:0) (red arrow/peak) isobars are illustrated. We confirmed ReTimeML assignments through secondary scans of the (**D**–**F**) d18:2 (*m/z* 262.3) and (**G**–**I**) d18:1 (*m/z* 264.3) sphingoid backbone. Differentiation of isotopic SLs was also supported by ReTimeML assignments for SM (d18:1/XX:1) isobars, as their secondary scans displayed interfering peaks of similar RT, caused by the presence of [M + 1] isotopic ions from HexCer (d18:1/XX:0) SLs. These were evidentially verified by the inclusion and alignment of the RTs for their (**J**–**L**) [M + H]^+^ MRM transitions. The isotopic distribution patterns for (**M**–**O**) SM(d18:1/18, 20 and 24:1) and (**P**–**R**) HexCer (d18:1/18, 20 and 24:1) have been presented to demonstrate how their profiles overlap, allowing the latter to interfere with [M + H]^+^  → *m/z* 264 scans for SM (d18:1/XX:1).

ReTimeML estimations help clarify the RTs of SLs where the occurrence of both isobaric and isotope distributions interferes with the correct peak identification in the total ion chromatogram (TIC), and cannot be resolved by supporting fragmentation data. This was particularly notable for di-unsaturated SMs that can correspond to either d18:1/XX:1 or d18:2/XX:0 in our MRM analyses, as the *m/z* 184 transition is insufficient to differentiate these isobars (Fig. 7A–C). To aid their resolution, secondary scanning of the *m/z* 262 (d18:2) and 264 (d18:1) sphingoid base was performed (Fig. 7D–I, Table 2). Sphingoid fragment selection proved useful in resolving SM(d18:2/XX:0) species (Fig. 7D–F) but presented complications in deriving the identities of SM (d18:1/XX:1) species, as the prominent peaks displayed with m/z 264 scans either did not align with MRET principles (Fig. 7G–H) or displayed multiple peaks, leading to further disambiguation of the correct identity (Fig. 7I). ReTimeML-guided RTs helped to either exclude (Fig. 7G,H) or correctly annotate (Fig. 7I) the SM (d18:1/XX:1) peaks in TICs. Peaks that did not align to ReTimeML estimates for *m/z* 264 transitions were attributed to isotope interference from mono-unsaturated hexosylceramides (HexCer (d18:1/XX:0), as illustrated (Fig. 7J–L). HexCer (d18:1/XX:0) species give rise to an [M + 1] isotopic ion that can interfere with the [M + H]^+^ ion of SM (d18:1/XX:1) (Fig. 7M–R). A similar comparison of the isotopic distribution and ReTimeML RT estimation helped to resolve SM (d18:2/XX:1) and SM (d18:1/XX:2), the latter of which were not assessed as their signal-to-noise ratios (S/N) were below the limit of detection (S/N < 3, data not shown).

### Correlation of Cer and SM profiles between CSF and serum

**Figure 8:**
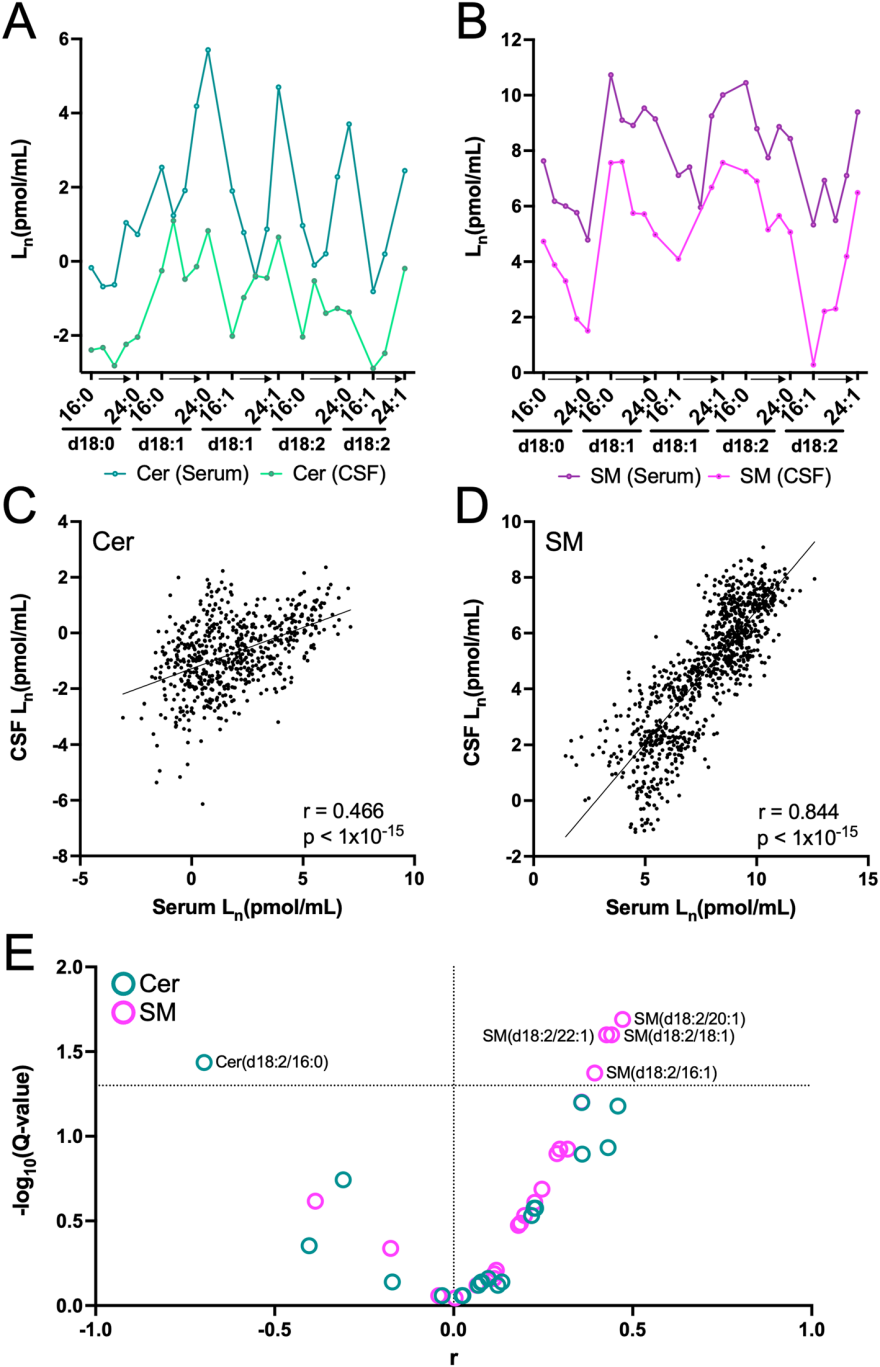
Human CSF-serum SL expression profiles for (**A**) Cer and (**B**) SM. (**C**,**D**) Scatter-plot of the individual pairwise CSF-serum SL comparisons for each (**C**) Cer (n = 606) and (**D**) SM (n = 945) identified in our subject cohort (n = 51). Correlations were determined by Pearson analysis, using natural log-transformed (Ln) Cer and SM levels. The coefficient of correlations (r) and *p* values are shown. (**E**) Volcano plot summarising the adjusted *p* values (Q-value) against correlation coefficients for Cer and SM lipids analysed. The dotted line shows the threshold for statistical significance at Q < 0.05, adjusted for multiple comparisons using the FDR approach of Benjamini, Krieger, and Yekutieli.

ReTimeML-guided Cer/SM lipids were assessed in our paired CSF and serum samples, with both fluids collected from the same HVs. This resulted in the structural characterisation and quantification of 48 Cer and SM lipid species. Demographics for the 51 HVs included in this analysis are described in Supplementary Table 1 and their concentrations (pmol/mL) for each Cer and SM lipid quantified in both the CSF and serum are listed in Supplementary Data 3. Participants were recruited in Cologne, Germany and considered healthy at the time of body fluid withdrawal, with no close relatives having a psychiatric disorder. Participants were comprised of 30 women (58.8%) and 21 men (41.2%), average age of 27.3 (SD = 6.6) years, BMI of 23.0 (SD = 3.4) and were of Caucasian ethnicity, with the exception of two participants of African and Asian origin. Cer and SM profiles were comparable to previously reported CSF and serum concentrations of these SLs in healthy/control cohorts^38–42^. SMs displayed predominately higher concentrations compared to Cer, with d18:1 being the most prominent sphingoid backbone for both SL classes, regardless of body fluid type (Fig. 8A,B, Supplementary Data 3).

The comparative similarities in their CSF-serum profiles (Fig. 8A,B) prompted us to evaluate the respective associations between these two body fluids. First, pairings were treated independently by matching every SL concentration identified per individual in the CSF to that of its corresponding value in the serum. This resulted in a total of 606 individualised Cer, and 945 SM, CSF-serum pairings (Fig. 8C,D), with Pearson analysis revealing significant (*p* < 1 × 10^−15^) positive associations between the biofluids for Cer (r = 0.466) and SM (r = 0.844).

Mean CSF-serum correlations for each characterised Cer and SM structure were evaluated next. From the 48 Cer and SM structures identified, 41 recorded a sufficient number of CSF-serum parings (N > 10) to be subjected to a correlated coefficient analysis (r), with statistical values (*p* values) adjusted for multiple comparisons using the FDR approach of Benjamini, Krieger, and Yekutieli (Q < 0.05). As a result, 5 SLs were defined as significantly correlating between the CSF and serum (Fig. 8E). Notably, all of these significant associations were comprised of noncanonical, sphingadiene backbone, with 4 SMs displaying positive associations (SM (d18:2/16:1), SM (d18:2/18:1), SM (d18:2/20:1) and SM (d18:2/22:1), alongside Cer (d18:2/16:0) which showed a significant inverse correlation between the two body fluids. Correlation coefficients and statistical values for all Cer and SM associations are summarised in Supplementary Table 2.

## Discussion

The objective of our study was to develop a user-friendly, analytically robust, tool for estimating RTs of the major d18:X SLs classes for Cer and SM, utilising our database of MRET behaviour in prior LC–MS/MS experiments, supplemented with literature sources, as the framework for building our model. Presently, no commercially available software or freeware incorporates RT data into their descriptor information to aid the identification of SLs in LC–MS/MS. However, it is strongly recommended that SL elution order be considered to reduce the likelihood of incorrect annotation in their identities^16^. The lack of RT incorporation is partly due to modelling overreliance on the specifics of the separation system used, with previous studies emphasising the need for future models to (1) be readily adaptable to differing experimental conditions and; (2) require as few reference values as possible^28,32,43^.

Applying MRET principles^35^, we demonstrate how ReTimeML is able to correctly assign various d18:0, d18:1 and d18:2 Cer/SM lipids based upon an understanding of their carbon chain length, degree of unsaturation and the C1 headgroup. We chose to focus on d18:X sphingoid bases as these comprise the most abundant SLs in mammalian organisms^44^. A similar approach has been developed for modelling the RTs of glycerophospholipids, assessing their equivalent carbon number (i.e., acyl chain composition), and expanding this across the different classes (e.g., phosphatidylcholine, phosphatidylethanolamine, phosphatidylserine)^45^. Both approaches have the distinct advantage of displaying no bias towards an MS-system (e.g., triple quadrupole (QqQ) or high-resolution orbitrap). This could have an added benefit for resolving Cer and SM lipids from global/untargeted LC–MS/MS analyses that employ the appropriate conditions for their separation^46^, particularly since high-resolution MS systems are without the added precision from MRM specifications to reduce TIC complexity. Though comparable, ReTimeML’s strength lies in its combination of learned material from previous experimental data, alongside the assignment of ‘real’ values (i.e., user-defined references, standards, internal controls) to enhance the decision-making process. This enabled ReTimeML to delineate common patterns for a given SL class, irrespective of LC–MS/MS methodology or experimental conditions, overcoming a major obstacle of RT adaptability for use in lipid identification software. Furthermore, ReTimeML is completely automated, making it accessible to those with limited knowledge of lipid biochemistry, and, thus, translatable to a base of researchers who may have shied away from the complex analyses.

Encouragingly, ReTimeML did not require an excessive number of references to estimate unknowns with a high degree of accuracy, meaning that users are not faced with the considerable costs of purchasing a copious number of standards. Moreover, for most experiments (excluding isocratic analyses) n ≥ 4 reference RTs did not drastically improve the accuracy of unknowns, and in certain instances increased deviations from user RTs, presumably due to data overfitting (Fig. 6). We recommend using a minimum of n = 3 reference points per SL class, be employed, comprised of different structural variants, as this provided the most accurate RT estimations (less than 1% variance for gradient and between 2.5 to 5.3% for isocratic, from user-defined measures, Fig. 6). ReTimeML’s lower accuracy of RT estimations in isocratic systems was attributed to the lesser data available for model training, as well as increases in peak-broadening from using single solvent systems that make analyte detection more difficult, particularly when lipids of interest span a wide polarity range or require the separation of closely related species^47,48^. Hence, isocratic measures are more suited to low numbers of SL analytes (n < 10) to reduce complexity but have the advantage of reduced run time with no requirement of column equilibration prior to subsequent measurements^49^.

For most SL analyses, n = 3 reference RTs would be achievable by pooling together a given LC–MS/MS experiments routine internal controls per lipid class (e.g., Cer, SM), or the closest structural/chemical equivalence, that control for lipid extraction inconsistencies and LC–MS/MS normalisation across sample cohorts^50,51^, alongside calibrators to externally quantify samples and/or QC mixture to control for instrument signal variability (e.g., ion suppression) and mitigate influences from matrix and batch effects during high throughput screening^52,53^.

Importantly, the accuracy of ReTimeML estimations was also adaptable to the reference material employed (Fig. 6), indicating that if a particular reference for a structure of interest (e.g., d18:2/XX:0) is not available, an alternate standard sourced from the same lipid class would suffice, provided our guidelines (n = 3 references, including structures) are retained. If standards cannot be procured, and we do acknowledge that certain classes of SLs (e.g., SM) are limited in their commercial availability, users can still choose to enter experimentally determined values from samples, though we strongly advise caution using this approach and recommend limiting this to RTs of SLs that are well-established or when signal interference is negligible to unequivocally resolve the correct RT on the TIC.

Herein we would also like to highlight ReTimeML’s proficiency at annotating RTs in complex TICs, particularly where ions of similar transitions interfere with correct peak annotation (Figs. 2 and 7), with their mass differentiation (~ 20 ppm) not achievable at the resolution of a QqQ system (~ 0.1–0.2 Da). ReTimeML correctly assigned RTs to the peaks of SM isobars (d18:1/XX:1 vs. d18:2/XX:0) using only the *m/z* 184 transition (Fig. 7A–C). This is particularly notable given this diagnostic ion is assigned only to detect the presence of a choline headgroup, with SM analyses requiring a secondary *m/z* 262, 264 or 266 scan(s) to determine the correct sphingoid backbone. This can be problematic in LC–MS/MS experiments for SMs as the choline headgroup is highly sensitive and its signal (up to 100× stronger) can suppress the detection of the sphingoid ion^54^, thereby limiting the structural information on SMs to the sum of its components (e.g., SM (36:2)) rather than at the fatty acyl/sphingoid base structure level (e.g., SM (d18:1/18:1))^55^. SM peak determination was further complicated by the presence of HexCer isotopic distribution, whose sphingoid transitions overlapped with di-unsaturated SMs (Figs. 7G–l). In the absence of RT annotation using ReTimeML, such signals could easily be misinterpreted, particularly if the *m/z* 184 was not in the MRM experiment or had already been assigned to a particular isobar (Fig. 7A,B).

Although a significant improvement in the resolution of SL variants, ReTimeML remains bound by the user’s LC setup. Should LC conditions not facilitate appropriate separation, interfering SL ions may be indistinguishable on the TIC. This has been proven to potentially cause artificial inflation of SL levels^36^, and represents a current constraint for ReTimeML to handle isomers of SLs (e.g., galactosyl vs glucosylceramide having been referred to as HexCer), given the chromatography conditions ReTimeML was trained on were incapable of their separation. Additionally, misidentifications from indistinguishable transitions of hydroxylated variants, even at trace levels, could artificiality inflate or cause miss-annotation (e.g., [M + H]^+^for Cer (d18:1/24:1) and [M + H–H_2_O]^+^ for Cer (t18:1/24:0) share the same 648.6 → 264.3 transition). Though not a component of this study, resolution of these hydroxylated variants can be achieved under suitable reverse-phase conditions^56^, nonetheless remain an important consideration when establishing LC conditions. Appropriate separation is also pivotal when considering the application of ReTimeML for processing SLs using high-resolution MS. As previously aforementioned, these untargeted measures provide no selective bias (i.e., MRM) towards SLs of interest, which could potentially increase the risk of overlapping or interfering ions from other lipid species. Updated versions of ReTimeML shall require training on additional setups that may circumvent these potential sources of interference, including normal-phase LC–MS/MS^25^ and next-generation ion mobility MS employing RT with collision cross-section^57^, capable of achieving additional SL class and structure interpretation.

ReTimeML was employed to aid the peak selection and structural annotation of Cer and SM lipids identified in HVs, providing both CSF and serum, allowing us to compare these body fluid profiles in the same subject. CSF is the closest anatomical fluid to the brain, likely to yield more applicable biomarkers for studying neuronal effects and conditions given its composition closely resembles that of the brain's extracellular space^58–60^. However, preconceived notions towards the invasiveness of the procedure (lumbar puncture) and the resulting distress to subjects have severely influenced its inclusion in clinical trials and broader use as a diagnostic fluid^61,62^. As SLs are enriched in the CNS and have exhibited a capacity to cross the blood–brain barrier^63^, their peripheral concentrations have the potential to act as surrogate markers in neurological and neuropsychiatric disorders^64^. To the best of our knowledge, this is the first reported CSF-serum SL comparison, and only the second paired blood-CSF lipid profiling investigation in HVs^65^.

Although Cer and SM concentrations in the CSF were found to be considerably lower than in serum, their relative structure-distribution patterns remained conserved and positively associated between the two body fluids (Fig. 8A–D). This is consistent with recently published CSF-plasma data^65^. Although Saito et al., reported contrasting results on overall lipid compositions, a closer inspection of their SM data revealed a similar conserved profile, with a positive correlation for the SM structural variants identified in their study (r = 0.811, average per lipid structure, n = 99; r = 0.760, individuals SL pairwise CSF-plasma pairings, n = 2,079; both *p* values < 0.0001, Supplementary Fig. 2). Their number of Cer identities was insufficient for an effective comparison (data not shown).

A handful of our SL identities exhibited highly stringent correlations, conserved between the CSF and serum (Q < 0.05, Fig. 8E). Interestingly, all the positively associated comparisons were categorised as belonging to the same SM structural variant, which included the presence of a sphingadiene backbone (d18:2, *m/z* 262), together with a mono-unsaturated fatty acyl chain. Though the presence of d18:2 on SLs was first identified in the late 60 s^66–68^, it has taken major advancements in LC–MS/MS sensitivity to enable routine assessments of these noncanonical structures, reviewed in^69^, meaning our understanding of their functional importance is largely undetermined. Sphingadiene backbones are exhibited in mouse kidney, brain, lung, and colon tissue SLs^70^, are reported to be the second most abundant sphingoid base in human plasma^71^, and a common constituent in plants and fungi^72,73^. Natural (soy) sphingadienes have been reported to inhibit intestinal tumoregensis in vivo, through disrupted Akt translocation^74^, and reduced Wnt transcriptional activity in colon cancer cells^75^. Clinical investigations have reported that d18:2 SLs may provide protection against the development of obesity and the risk of diabetes and cardiovascular disease^76,77^. Our own research observed a marked accumulation of d18:2 SLs (unpublished), following the ablation of sphingosine kinase 2 (SphK2) in a mouse model for Alzheimer’s disease (J20), which led to severe defects in myelin integrity^78^. However, we never resolved whether this shift towards sphingadiene-based SLs was a primary cause of myelin disruption. SphK2 is the major isoform for catalysing the phosphorylation of sphingosine into sphingosine 1-phosphate (S1P), the penultimate step in SL lysosomal catabolism via irreversible degradation by S1P lyase^1,2^. This pathway has been reported to be less efficient for the clearance of d18:2 SLs, over their d18:1 counterparts in vitro^4,70^, presumably a consequence of the angled nature of the cis-double bond^79^. Furthermore, it has been recently shown that d18:2 predominately converts to SMs over glycosphingolipids (i.e., HexCer)^79^. Hence, we speculate that stronger associations observed for SM (d18:2/XX:1) profiles between these peripheral systems may be rationalised by their preferential formation from sphingadiene precursors and the accompanied stability from cis-double bonds on both the sphingoid base and fatty acyl chain.

In this study, we set ourselves the objective of developing a freeware that could perform data transformation and feature engineering to estimate the RTs of Cer and SM identities from complex LC–MS/MS spectra, adaptable to the experimental conditions applied, and amenable to any level of LC–MS/MS experience and/or knowledge on the biochemistry of SLs. We believe that ReTimeML excelled at this objective, assisting with the identification process across multiple LC–MS/MS conditions, including structural annotation and the removal of interfering RTs from isobaric and isotopic measures. While we recognise the advantages from employing ReTimeML, we acknowledge the existence of unforeseen circumstances and have highlighted probable instances where ReTimeML may not be applicable to a user’s LC–MS/MS design. Hence, it is always advisable for users to conduct secondary analyses/measures to confirm their SL identities, particularly if the existence of isobaric compounds or ion interferences within the TIC are likely, albeit not directly observed.

Moving forward, our objective is the continued optimisation of ReTimeML, ensuring it grows with mass spectrometry development and refine its ability to guide RT annotations for further classes of SLs, including non-canonical variants. We also plan to expand ReTimeML assessments into other lipid classes, searching relevant data repositories (e.g., MetaboLights, Metabolomics Work Bench), and welcome support from users prepared to share their lipidomic data via the options provided (see ‘Data and Code availability’). In the long term, we envisage that with the incorporation of more machine-learned lipid class RT measures, ReTimeML could become an openly accessed and/or integrated function in current automated lipid identification software engines. In achieving routine RT annotations for SLs, we also drew attention to the physiological and pathophysiological importance of non-canonical SLs that are achievable with current mass spectrometric systems. It is hoped that our findings will further scrutinise their significance as molecular mediators in health and disease.

## Materials and methods

### Data integration

Regression models were fitted to Cer and SM data collated from our prior published material and that of the literature (Supplementary Data 2). Data incorporated met a minimum quantity of molecular and chromatography information, ensuring sufficient variance between descriptors to accurately fit with models. The information incorporated includes the sphingoid base/fatty-acyl naming for Cer and SM^55,80^ and/or chemical formula of the sphingolipid, *m/z* of the [M + H]^+^ precursor ion together, with a minimum of one fragmentation ion to aid the structural characterisation (e.g., *m/z* 264 for the sphingosine backbone), the chromatography system applied, stationary-phase column used, solvent conditions (gradient vs isocratic) and nominated flow rate. For each dataset, Cer and SM species were broken down into RTs that were either “user-defined” or “known” (e.g., standard), per study. The selected nominations were defined by the experimental datasets chosen controls (internal and QC), compounds used for calibration or to optimise the LC–MS/MS parameters during their method development.

### Retention time learning algorithms

Linear and non-linear regression algorithms were assessed and evaluated based on their ability to predict RTs, alongside the required number of training samples to learn and achieve appropriate levels of accuracy. Variables associated with precursor mass, precursor mass squared, square root and the log of the precursor mass for each molecular sample were calculated for every included data point. Adopting the sphingoid base and fatty acyl notation^55^, a Python (version 3.10, Python Software Foundation) function was used to extract the molecular features, based on the nominated Cer/SM precursor mass and relevant fragmentation to deduce the carbon chain length, both on the sphingoid backbone and fatty acyl chain, the degree(s) of unsaturation, as well as specific modifications to the C1 head group (i.e., *m/z* 184 for choline phosphate of SM). Incorporated datasets were randomly divided into ‘training’ (70%) and ‘validation’ (30%), using Python’s inbuilt sklearn package, which was also used to train linear, lasso and ridge regression models. Python’s xgboost package was used to train an XGBoost regression algorithm. RMSE and R^2^ were used to evaluate the performance of each regression model’s RT estimations. The number of training, and hence corresponding validation data points were sequentially varied (± 3 datasets) to assess the minimum training size required for a given regression model to forecast RTs accurately (R^2^ > 0.95; RMSE < 0.25), with regression models ranked according to the number of training samples required to achieve these measurables. The complete list of regression models, along with R^2^ and RMSE scores per training size, are summarised in Supplementary Data 1.

### Ceramide and sphingomyelin data acquisition

Top-ranked regression models for Cer and SM were selected for secondary verification across four, independently performed, LC–MS/MS assessments spanning human fluids (CSF and serum), mouse liver and rat brain homogenates (all unpublished analyses). Human CSF and serum were provided by our biobank at the Central Institute of Mental Health, Mannheim, with both donated from the same healthy participants (n = 51), originally recruited at the Clinic and Outpatient Clinic of Psychiatry and Psychotherapy, University of Cologne. The Ethics Committee of the Medical Faculty Cologne, University of Cologne, Germany (00-053) approved the use of these samples for this research. Rat brain tissue was procured from our prior animal investigation exploring behavioural changes following different tetrahydrocannabinol preparations^81^, approved by the regional authority State Agency for Nature, Environment and Consumer Protection of the State North Rhine-Westphalia (LANIUV-NRW). Only tissue from placebo-administered rats was assessed. SLs extracted from C57BL/6 mouse liver were in accordance with protocols (#2019-033), approved by the Research Ethics and Governance Office, Royal Prince Alfred Hospital, Sydney, Australia.

SLs from human fluids were extracted using the conventional Bligh and Dyer method^82^, while rodent liver and brain tissue SLs were extracted using single-phase methanol/butanol (1:1 v/v^83^) and two-phase methyl-tert-butyl ether (MTBE)/methanol/water (10:3:2.5, v/v/v^4^), respectively. Prior to extractions, all samples were loaded with Cer (d18:1/17:0) and SM (d18:1/12:0) as internal controls. All samples underwent MRM analysis performed on a TSQ Altis QqQ mass spectrometer (ThermoFisher), coupled to a Vanquish UHPLC system, as previous^4^. For SMs, secondary product ion scans for *m/z* 264 and 262 were included to help distinguish isobars (e.g., SM(d18:1/XX:1) and SM (d18:2/XX:0)) and the potentially conflicting phosphatidylcholine ions, which induce the same *m/z* 184 choline headgroup fragment. A complete list of Cer and SM lipids, together with their MRM transitions, are provided in Tables 1 and 2.

Chromatographic and stationary phase conditions were also varied between experiments. SLs extracted from human fluids were resolved on a 3 × 150 mm Agilent XDB-C8 column (5 μM pore size), using a modified Hejazi et al.^35^ binary gradient as follows: 0 min, 20:80 A/B; 2 min, 20:80 A/B; 7 min, 13:87 A/B; 14 min, 0:100 A/B; 20.5 min, 0:100 A/B; 21 min, 20:80 A/B; 24 min, 20:80 A/B. Mobile phase ‘A’ consisted of 0.2% formic acid, 2 mM ammonium formate in water; Mobile phase ‘B’: 0.2% formic acid, 1 mM ammonium formate in methanol. Total run time was 24 min, at a flow rate of 0.2 mL/min. Extracted SLs from mouse liver were separated on the same 3 × 150 mm Agilent XDB-C8 stationary phase, with modified gradient solvents and conditions as follows: 0 min, 20:80 A/B; 1 min, 20:80 A/B; 9 min, 5:95 A/B; 11 min, 0:100 A/B; 17.5 min, 0:100 A/B; 17.6 min 20:80 A/B, 20.5 min 20:80 A/B. Solvent ‘A’ comprised of 0.1% formic acid, 2 mM Ammonium acetate in water; Solvent ‘B’ composition of 0.1% formic acid, 2 mM Ammonium acetate in methanol. Total run time was 20.5 min at a flow rate of 0.3 mL/min. SL analysis of brains (rat) were resolved on a 2.1 × 100 mm Waters Acquity C18 UPLC column (1.7 µm pore size) under isocratic conditions, as previous^84^, using methanol with 0.2% formic acid as the mobile phase, at a flow rate of 0.25 mL/min for 15 min.

### ReTimeML interface

ReTimeML’s pilot version is available as a free, open-source web interface powered by streamlit (https://mikeallwright23-retime-app-lipid3-021zpv.streamlit.app/). Users upload datasets in .csv format, consisting of the Cer/SM lipids of interest, alongside their precursor mass and whether the SL species included are a “Train” (reference with known RT included) or “Test” (unknown) value. Template .csv files are provided for users to test the interface (Supplementary Data 4 and 5), and can be adapted for their own SL analysis. Uploaded data (drag and drop option) triggers the automatic calculation of RTs, utilising the nominated RTs as points-of-reference (Train) to guide the extrapolated unknowns (Test), with users free to amend the number of train/test values. It is of note to mention a minimum of two ‘Train’ values is required for ReTimeML to extrapolate an output. Functions are applied within the web interface to automatically pre-process each data field, using regex functionality in Python, to feature engineer the number of carbon atoms and degree(s) of unsaturation for structural components on the sphingoid and fatty acyl chain, programming these as one hot encoded (ohe) variables, as well as the log of the mass, mass squared and square root of the mass. In addition, ReTimeML also provides an MRET profile output of estimations, annotated using second-order polynomial trendlines. The web interface also provides users the added option to voluntarily upload their own RT estimations, so that our team can evaluate and incorporate them into our working models.

### Statistical analysis

Verified Human Cer and SM lipid datasets underwent peak integration using Xcalibur 4.4.16.14 software (ThermoFisher Scientific, San Jose, CA, USA), with Cer and SM species normalised as ratios to their class-specific internal control. A separate Cer/SM mixture, comprising various compounds (Supplementary Table 3), was run every 20 samples. This external mixture acted as a QC and provided additional RT references for Cer and SM. All Cer/SM mixtures were prepared in aqueous/organic proportions reflecting the starting conditions of the respective LC–MS/MS experiments. All Cer and SM values were first log-transformed (natural log) to obtain a normal distribution for Pearson correlations (r). For the assessment of individual lipids between CSF and serum, the resultant *p* values were adjusted for multiple comparisons using the Benjamini, Krieger, and Yekutieli false discovery rate (FDR) approach, with Q < 0.05 considered significant (GraphPad Prism software, Version 10.0.3, Dotmatics, Boston, MA, USA).

### Supplementary Information

Supplementary Information.

Supplementary Legends.

Dataset S1.

Dataset S2.

Dataset S3.

Dataset S4.

Dataset S5.

## Data Availability

All Cer and SM data supporting our findings has been provided in the manuscript and supplementary information. Datasets for ReTimeML training were collated from prior published material. Details for these studies, including the Cer and SM lipids assessed, has been provided in Supplementary Data 2. RT values for these studies can be made available by request to the corresponding authors. Source code for ReTimeML learned regression models is also available upon reasonable request. We encourage users of ReTimeML to voluntarily upload RT data for future lipid regression model development (.xls, .xlsx or .csv format), at either the following Google form https://forms.gle/p9fGuzuujZqDhcpD6 or Dropbox link: https://www.dropbox.com/request/kMOXwBe54IxGWeocE7WS.

## References

[1] Hannun YA, Obeid LM (2018). Sphingolipids and their metabolism in physiology and disease. Nat. Rev. Mol. Cell Biol..

[2] Merrill AH (2011). Sphingolipid and glycosphingolipid metabolic pathways in the era of sphingolipidomics. Chem. Rev..

[3] Giussani P, Prinetti A, Tringali C (2021). The role of Sphingolipids in myelination and myelin stability and their involvement in childhood and adult demyelinating disorders. J. Neurochem..

[4] Couttas TA, Rustam YH, Song H (2020). A novel function of sphingosine kinase 2 in the metabolism of sphinga-4,14-diene lipids. Metabolites.

[5] Goni FM, Alonso A (2006). Biophysics of sphingolipids I. Membrane properties of sphingosine, ceramides and other simple sphingolipids. Biochim. Biophys. Acta.

[6] Hannun YA, Bell RM (1989). Functions of sphingolipids and sphingolipid breakdown products in cellular regulation. Science.

[7] van Meer G, Voelker DR, Feigenson GW (2008). Membrane lipids: Where they are and how they behave. Nat. Rev. Mol. Cell Biol..

[8] Fanani ML, Maggio B (2017). The many faces (and phases) of ceramide and sphingomyelin I - single lipids. Biophys. Rev..

[9] Taniguchi M, Okazaki T (2014). The role of sphingomyelin and sphingomyelin synthases in cell death, proliferation and migration-from cell and animal models to human disorders. Biochim. Biophys. Acta.

[10] Maceyka M, Spiegel S (2014). Sphingolipid metabolites in inflammatory disease. Nature.

[11] Kolesnick R (2002). The therapeutic potential of modulating the ceramide/sphingomyelin pathway. J. Clin. Investig..

[12] Ryan E, Nguyen CQN, Shiea C (2017). Detailed structural characterization of sphingolipids via 193 nm ultraviolet photodissociation and ultra high resolution tandem mass spectrometry. J. Am. Soc. Mass Spectrom..

[13] Bielawski J, Szulc ZM, Hannun YA (2006). Simultaneous quantitative analysis of bioactive sphingolipids by high-performance liquid chromatography-tandem mass spectrometry. Methods.

[14] Couttas TA, Kain N, Tran C (2018). Age-dependent changes to sphingolipid balance in the human hippocampus are gender-specific and may sensitize to neurodegeneration. J. Alzheimers Dis..

[15] Sullards MC, Merrill AH (2001). Analysis of sphingosine 1-phosphate, ceramides, and other bioactive sphingolipids by high-performance liquid chromatography-tandem mass spectrometry. Sci. STKE.

[16] Peng B, Kopczynski D, Pratt BS (2020). LipidCreator workbench to probe the lipidomic landscape. Nat. Commun..

[17] Koelmel JP, Kroeger NM, Ulmer CZ (2017). LipidMatch: an automated workflow for rule-based lipid identification using untargeted high-resolution tandem mass spectrometry data. BMC Bioinform..

[18] Wong JW, Abuhusain HJ, McDonald KL (2012). MMSAT: Automated quantification of metabolites in selected reaction monitoring experiments. Anal. Chem..

[19] Merrill AH, Sullards MC (2017). Opinion article on lipidomics: Inherent challenges of lipidomic analysis of sphingolipids. Biochim. Biophys. Acta Mol. Cell Biol. Lipids.

[20] Zullig T, Kofeler HC (2021). High resolution mass spectrometry in lipidomics. Mass Spectrom. Rev..

[21] Duan J, Merrill AH (2015). 1-Deoxysphingolipids encountered exogenously and made de novo: Dangerous mysteries inside an enigma. J. Biol. Chem..

[22] Sandhoff R (2010). Very long chain sphingolipids: Tissue expression, function and synthesis. FEBS Lett..

[23] Merrill AH, Sullards MC, Allegood JC (2005). Sphingolipidomics: high-throughput, structure-specific, and quantitative analysis of sphingolipids by liquid chromatography tandem mass spectrometry. Methods.

[24] Sullards MC, Liu Y, Chen Y (2011). Analysis of mammalian sphingolipids by liquid chromatography tandem mass spectrometry (LC-MS/MS) and tissue imaging mass spectrometry (TIMS). Biochim. Biophys. Acta.

[25] Zama K, Hayashi Y, Ito S (2009). Simultaneous quantification of glucosylceramide and galactosylceramide by normal-phase HPLC using O-phtalaldehyde derivatives prepared with sphingolipid ceramide N-deacylase. Glycobiology.

[26] Rana NA, Singh A, Del Poeta M, Hannun YA (2015). Qualitative and quantitative measurements of sphingolipids by mass spectrometry. Bioactive Sphingolipids in Cancer Biology and Therapy.

[27] Osipenko S, Bashkirova I, Sosnin S (2020). Machine learning to predict retention time of small molecules in nano-HPLC. Anal. Bioanal. Chem..

[28] Stanstrup J, Neumann S, Vrhovsek U (2015). PredRet: Prediction of retention time by direct mapping between multiple chromatographic systems. Anal. Chem..

[29] D'Archivio AA, Giannitto A, Maggi MA (2012). Cross-column retention prediction in reversed-phase high-performance liquid chromatography by artificial neural network modelling. Anal. Chim. Acta.

[30] Fedorova ES, Matyushin DD, Plyushchenko IV (2022). Deep learning for retention time prediction in reversed-phase liquid chromatography. J. Chromatogr. A.

[31] Yang Q, Ji H, Lu H (2021). Prediction of liquid chromatographic retention time with graph neural networks to assist in small molecule identification. Anal. Chem..

[32] Aicheler F, Li J, Hoene M (2015). Retention time prediction improves identification in nontargeted lipidomics approaches. Anal. Chem..

[33] Bouwmeester R, Martens L, Degroeve S (2019). Comprehensive and empirical evaluation of machine learning algorithms for small molecule LC retention time prediction. Anal. Chem..

[34] Domingo-Almenara X, Guijas C, Billings E (2019). The METLIN small molecule dataset for machine learning-based retention time prediction. Nat. Commun..

[35] Hejazi L, Wong JW, Cheng D (2011). Mass and relative elution time profiling: two-dimensional analysis of sphingolipids in Alzheimer's disease brains. Biochem. J..

[36] Huang H, Tong TT, Yau LF (2018). LC–MS based sphingolipidomic study on A549 human lung adenocarcinoma cell line and its taxol-resistant strain. BMC Cancer.

[37] Huynh K, Barlow CK, Jayawardana KS (2019). High-throughput plasma lipidomics: Detailed mapping of the associations with cardiometabolic risk factors. Cell Chem. Biol..

[38] Fonteh AN, Ormseth C, Chiang J (2015). Sphingolipid metabolism correlates with cerebrospinal fluid beta amyloid levels in Alzheimer's disease. PLoS ONE.

[39] Hammad SM, Harden OC, Wilson DA (2021). Plasma sphingolipid profile associated with subclinical atherosclerosis and clinical disease markers of systemic lupus erythematosus: potential predictive value. Front. Immunol..

[40] Hanamatsu H, Ohnishi S, Sakai S (2014). Altered levels of serum sphingomyelin and ceramide containing distinct acyl chains in young obese adults. Nutr. Diabetes.

[41] Hammad SM, Pierce JS, Soodavar F (2010). Blood sphingolipidomics in healthy humans: Impact of sample collection methodology. J. Lipid Res..

[42] Torretta E, Arosio B, Barbacini P (2018). Particular CSF sphingolipid patterns identify iNPH and AD patients. Sci. Rep..

[43] Ross DH, Guo J, Bilbao A (2023). Evaluating software tools for lipid identification from ion mobility spectrometry-mass spectrometry lipidomics data. Molecules.

[44] Carreira AC, Santos TC, Lone MA (2019). Mammalian sphingoid bases: Biophysical, physiological and pathological properties. Prog. Lipid Res..

[45] White JB, Trim PJ, Salagaras T (2022). Equivalent carbon number and interclass retention time conversion enhance lipid identification in untargeted clinical lipidomics. Anal. Chem..

[46] Marian OC, Teo JD, Lee JY (2023). Disrupted myelin lipid metabolism differentiates frontotemporal dementia caused by GRN and C9orf72 gene mutations. Acta Neuropathol. Commun..

[47] Gritti F (2016). General theory of peak compression in liquid chromatography. J. Chromatogr. A.

[48] Haidar Ahmad IA (2017). Necessary analytical skills and knowledge for identifying, understanding, and performing HPLC troubleshooting. Chromatographia.

[49] Schellinger AP, Carr PW (2006). Isocratic and gradient elution chromatography: A comparison in terms of speed, retention reproducibility and quantitation. J. Chromatogr. A.

[50] Holcapek M, Liebisch G, Ekroos K (2018). Lipidomic analysis. Anal. Chem..

[51] Kofeler HC, Ahrends R, Baker ES (2021). Recommendations for good practice in MS-based lipidomics. J. Lipid Res..

[52] Pitt JJ (2009). Principles and applications of liquid chromatography–mass spectrometry in clinical biochemistry. Clin. Biochem. Rev..

[53] Cheng WL, Markus C, Lim CY (2023). Calibration practices in clinical mass spectrometry: Review and recommendations. Ann. Lab. Med..

[54] Hsu FF, Turk J (2000). Structural determination of sphingomyelin by tandem mass spectrometry with electrospray ionization. J. Am. Soc. Mass Spectrom..

[55] Liebisch G, Vizcaino JA, Kofeler H (2013). Shorthand notation for lipid structures derived from mass spectrometry. J. Lipid Res..

[56] Hartler J, Armando AM, Trotzmuller M (2020). Automated annotation of sphingolipids including accurate identification of hydroxylation sites using MS(n) data. Anal. Chem..

[57] Camunas-Alberca SM, Moran-Garrido M, Saiz J (2023). Integrating the potential of ion mobility spectrometry–mass spectrometry in the separation and structural characterisation of lipid isomers. Front. Mol. Biosci..

[58] Blennow K, Hampel H, Weiner M (2010). Cerebrospinal fluid and plasma biomarkers in Alzheimer disease. Nat. Rev. Neurol..

[59] Lee J (2022). Cerebrospinal fluid biomarkers in various pediatric neurologic diseases. Clin. Exp. Pediatr..

[60] Paraskevas GP (2022). The role of cerebrospinal fluid biomarkers in dementia and other related neurodegenerative disorders. Brain Sci.

[61] Howell JC, Parker MW, Watts KD (2016). Research lumbar punctures among African Americans and Caucasians: Perception predicts experience. Front. Aging Neurosci..

[62] Day GS, Rappai T, Sathyan S (2020). Deciphering the factors that influence participation in studies requiring serial lumbar punctures. Alzheimers Dement. (Amst.).

[63] de la Monte SM (2012). Triangulated mal-signaling in Alzheimer's disease: Roles of neurotoxic ceramides, ER stress, and insulin resistance reviewed. J. Alzheimers Dis..

[64] van Kruining D, Luo Q, van Echten-Deckert G (2020). Sphingolipids as prognostic biomarkers of neurodegeneration, neuroinflammation, and psychiatric diseases and their emerging role in lipidomic investigation methods. Adv. Drug Deliv. Rev..

[65] Saito K, Hattori K, Hidese S (2021). Profiling of cerebrospinal fluid lipids and their relationship with plasma lipids in healthy humans. Metabolites.

[66] Renkonen O, Hirvisalo EL (1969). Structure of plasma sphingadienine. J. Lipid Res..

[67] Polito AJ, Akita T, Sweeley CC (1968). Gas chromatography and mass spectrometry of sphingolipid bases. Characterization of sphinga-4,14-dienine from plasma sphingomyelin. Biochemistry.

[68] Panganamala RV, Geer JC, Cornwell DG (1969). Long-chain bases in the sphingolipids of atherosclerotic human aorta. J. Lipid Res..

[69] Lam BWS, Yam TYA, Chen CP (2021). The noncanonical chronicles: Emerging roles of sphingolipid structural variants. Cell. Signal.

[70] Jojima K, Edagawa M, Sawai M (2020). Biosynthesis of the anti-lipid-microdomain sphingoid base 4,14-sphingadiene by the ceramide desaturase FADS3. FASEB J..

[71] Karsai G, Lone M, Kutalik Z (2020). FADS3 is a Delta14Z sphingoid base desaturase that contributes to gender differences in the human plasma sphingolipidome. J. Biol. Chem..

[72] Sullards MC, Lynch DV, Merrill AH (2000). Structure determination of soybean and wheat glucosylceramides by tandem mass spectrometry. J. Mass Spectrom..

[73] Aida K, Kinoshita M, Tanji M (2005). Prevention of aberrant crypt foci formation by dietary maize and yeast cerebrosides in 1, 2-dimethylhydrazine-treated mice. J. Oleo Sci..

[74] Fyrst H, Oskouian B, Bandhuvula P (2009). Natural sphingadienes inhibit Akt-dependent signaling and prevent intestinal tumorigenesis. Cancer Res..

[75] Kumar A, Pandurangan AK, Lu F (2012). Chemopreventive sphingadienes downregulate Wnt signaling via a PP2A/Akt/GSK3beta pathway in colon cancer. Carcinogenesis.

[76] Chew WS, Torta F, Ji S (2019). Large-scale lipidomics identifies associations between plasma sphingolipids and T2DM incidence. JCI Insight.

[77] Othman A, Saely CH, Muendlein A (2015). Plasma C20-Sphingolipids predict cardiovascular events independently from conventional cardiovascular risk factors in patients undergoing coronary angiography. Atherosclerosis.

[78] Lei M, Teo JD, Song H (2019). Sphingosine kinase 2 potentiates amyloid deposition but protects against hippocampal volume loss and demyelination in a mouse model of Alzheimer's disease. J. Neurosci..

[79] Jojima K, Kihara A (2023). Metabolism of sphingadiene and characterization of the sphingadiene-producing enzyme FADS3. Biochim. Biophys. Acta Mol. Cell Biol. Lipids.

[80] Fahy E, Subramaniam S, Murphy RC (2009). Update of the LIPID MAPS comprehensive classification system for lipids. J. Lipid Res..

[81] Rohleder C, Pahlisch F, Graf R (2020). Different pharmaceutical preparations of Delta(9)-tetrahydrocannabinol differentially affect its behavioral effects in rats. Addict. Biol..

[82] Bligh EG, Dyer WJ (1959). A rapid method of total lipid extraction and purification. Can. J. Biochem. Physiol..

[83] Alshehry ZH, Barlow CK, Weir JM (2015). An efficient single phase method for the extraction of plasma lipids. Metabolites.

[84] Turner N, Lim XY, Toop HD (2018). A selective inhibitor of ceramide synthase 1 reveals a novel role in fat metabolism. Nat. Commun..

