# Supplementary material for: ReTimeML: a retention time predictor that supports the LC–MS/MS analysis of sphingolipids
Source: Sci Rep. 2024 Feb 22;14:4375. doi: 10.1038/s41598-024-53860-0 (PMC10883992; doi:10.1038/s41598-024-53860-0)
Supplement: Supplementary file 1 — Supplementary Information. [file 41598_2024_53860_MOESM1_ESM.pdf]

Supplementary Information for

ReTimeML: A retention time predictor that supports the LC-MS/MS analysis of sphingolipids.

Michael Allwright *et al.*

*Corresponding author: Timothy Couttas, Email: timothy.couttas@sydney.edu.au

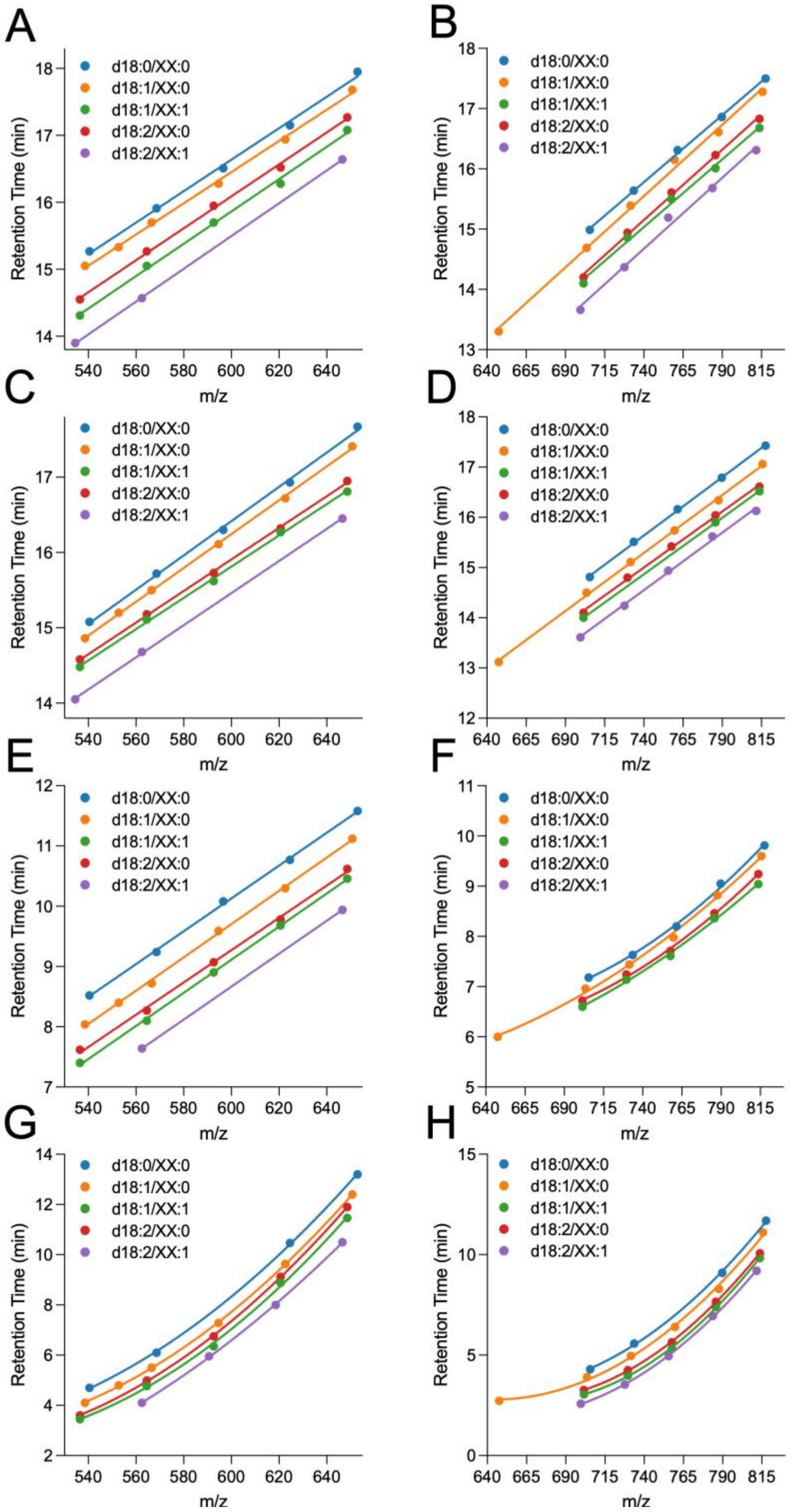

Supplementary Figure 1. 2D plots of *m/z* vs user-defined RTs across the four validation LC-MS/MS analyses of ceramide (A, C, E, G) and SM (B, D, F, H) content from (A, B) human serum, (C, D) human CSF, (E, F) mouse liver and (G, H) rat brain tissue.

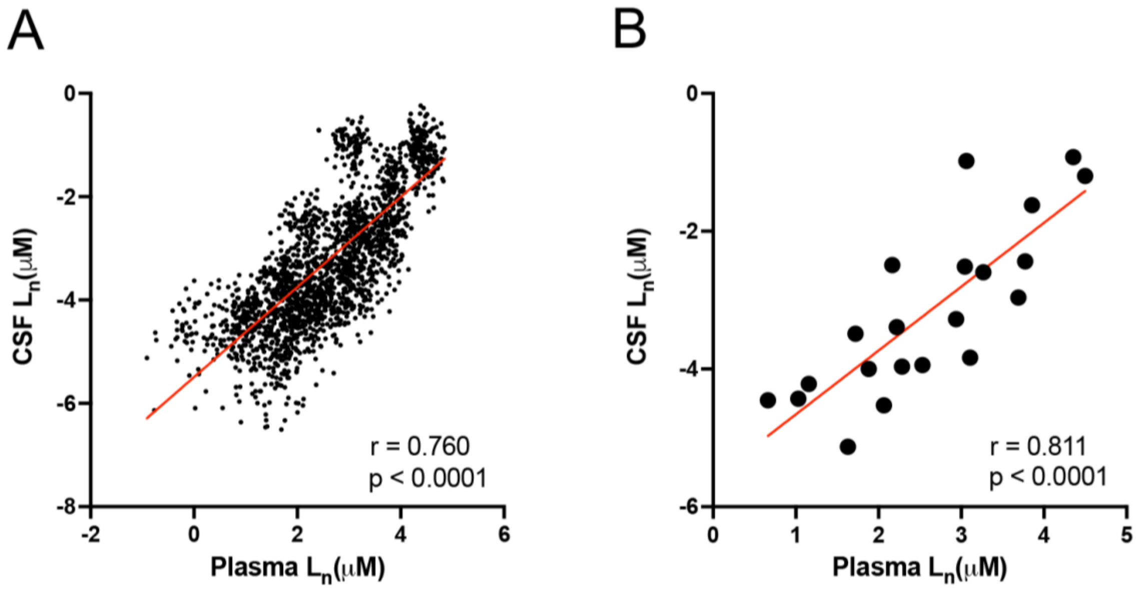

Supplementary Figure 2. Scatter plots representing (A) individual and (B) average pairwise CSF-plasma comparisons for SMs identified by Saito *et al.*, [63]. The coefficient of correlations (r) and p-value are shown, with correlations determined by Pearson analysis using natural log-transformed data to achieve normal distribution.

Supplementary Table 1. Participant demographics.

ID	Age	Gender	Occupation	Height (m)	Weight (kg)	BMI (kg/m2)	Ethnicity	Marital status	Number of kids
64	36	female	NA	1.65	51	18.73	Caucasian	ND	NA
65	49	female	NA	1.68	53	18.78	African	ND	NA
78	25	female	Student (Medicine)	1.75	64	20.90	Caucasian	single	0
79	39	male	NA	NA	NA	NA	Caucasian	ND	NA
81	27	male	Student (Medicine, Sports)	1.80	75	23.15	Caucasian	single	0
82	21	male	Student (Law)	NA	NA	NA	Caucasian	single	0
88	23	male	Student (Medicine)	1.80	70	21.60	Caucasian	ND	NA
89	22	female	Student (Medicine)	1.75	68	22.20	Caucasian	single	0
92	32	female	Nurse	1.74	74	24.44	Caucasian	single	1
93	35	male	Student (Medicine)	1.79	80	24.97	Caucasian	single	0
100	22	female	Student (Medicine, Sports)	1.73	57	19.05	Caucasian	single	0
102	23	female	Student (Medicine)	1.81	64	19.54	Caucasian	single	0
107	19	female	Student (Secondary School)	1.63	49	18.44	Caucasian	single	0
108	19	male	Apprenticeship office clerk	1.75	100	32.65	Caucasian	single	0
109	27	male	Student (Medicine)	1.80	73	22.53	Caucasian	single	0
111	40	female	Housewife	1.65	53	19.47	Caucasian	married	3
112	44	male	Chemical engineer	1.70	93	32.18	Caucasian	married	3
113	28	female	Nurse	1.65	70	25.71	Caucasian	married	0
116	27	male	Nurse	1.93	90	24.16	Caucasian	single	0
117	21	female	Student	1.70	73	25.26	Caucasian	single	0
119	38	female	Nursing assistant	1.70	63	21.80	Caucasian	married	3
121	30	male	NA	1.85	75	21.91	Caucasian	ND	NA
123	29	female	Physician	1.67	61	21.87	Caucasian	ND	0
124	24	female	Student (Medicine)	1.65	55	20.20	Caucasian	single	0
125	23	male	Student (Medicine)	1.87	110	31.46	Caucasian	single	0
128	22	female	Student (Medicine)	1.70	55	19.03	Caucasian	single	0
130	23	male	Student	1.73	57	19.05	Asian	single	0
135	34	male	Nurse	1.82	86	25.96	Caucasian	single	0
140	24	female	Nurse	1.67	62	22.23	Caucasian	single	0
141	26	female	Student (Medicine)	1.62	68	25.91	Caucasian	single	0
142	21	female	Student (Biology, Sports)	1.70	63	21.80	Caucasian	single	0
143	23	female	Student (Geography, Ethnology)	1.73	70	23.39	Caucasian	single	0
145	26	female	Nurse	1.70	70	24.22	Caucasian	single	0
146	27	female	Student (German Studies, Sociology)	1.68	65	23.03	Caucasian	single	0
149	29	male	Student (Medicine)	1.82	80	24.15	Caucasian	single	0
150	28	female	insurance saleswoman	1.65	65	23.88	Caucasian	single	0
151	29	male	Nurse	1.82	70	21.13	Caucasian	single	0
160	29	female	Physician	1.64	52	19.33	Caucasian	single	0
161	22	female	Student (Medicine)	1.81	79	24.11	Caucasian	single	0
169	30	male	Student (Mechanical Engineer)	1.93	85	22.82	Caucasian	single	0
170	23	female	Studying (Medicine)	1.65	58	21.30	Caucasian	single	0
171	28	male	Nurse	1.70	72	24.91	Caucasian	married	0
207	24	male	Studying (Medicine)	1.78	70	22.09	Caucasian	single	0
211	18	female	Student (Secondary School)	1.75	60	19.59	Caucasian	single	0
1012	28	female	Child and adolescent psychotherapist	1.61	68	26.23	Caucasian	single	0
1020	23	female	Student	1.70	86	29.76	Caucasian	single	0
1075	26	male	Medical student	1.88	86	24.33	Caucasian	single	0
1080	23	male	Student	1.87	90	25.74	Caucasian	single	0
1081	23	female	Student	1.75	55	17.96	Caucasian	single	0
1089	25	male	Student	1.79	70	21.85	Caucasian	single	0
1095	37	female	Physician	1.75	72	23.51	Caucasian	single	0

List of abbreviations: NA, Not assessed; ND, Preferred not to disclose.

Supplementary Table 2. Ceramide and sphingomyelin correlations between the CSF and serum of HVs.

	**Ceramide**			**Sphingomyelin**		
**Structure**	**r**[1]	**p-value**[2]	**Q-value**[3]	**r**[1]	**p-value**[2]	**Q-value**[3]
d18:0/16:0	0.124	0.702	0.701	0.296	0.035	0.095
d18:0/18:0	-0.404	0.281	0.412	0.187	0.190	0.299
d18:0/20:0	0.026	0.919	0.818	0.119	0.425	0.562
d18:0/22:0	-0.171	0.615	0.662	-0.176	0.304	0.430
d18:0/24:0	ND	ND	ND	0.065	0.691	0.701
d18:1/16:0	0.023	0.904	0.818	0.358	0.010	0.051
d18:1/18:0	0.097	0.512	0.621	0.198	0.163	0.268
d18:1/20:0	0.218	0.156	0.265	0.246	0.081	0.176
d18:1/22:0	0.224	0.134	0.238	0.226	0.110	0.215
d18:1/24:0	0.357	0.012	0.054	0.181	0.204	0.310
d18:1/16:1	ND	ND	ND	ND	ND	ND
d18:1/18:1	-0.309	0.067	0.153	ND	ND	ND
d18:1/20:1	0.069	0.723	0.705	ND	ND	ND
d18:1/22:1	0.076	0.637	0.669	ND	ND	ND
d18:1/24:1	0.229	0.131	0.238	0.004	0.978	0.834
d18:2/16:0	-0.697	0.004	**0.020***	0.113	0.464	0.593
d18:2/18:0	0.359	0.044	0.106	0.318	0.033	0.095
d18:2/20:0	0.431	0.028	0.088	-0.386	0.102	0.210
d18:2/22:0	0.459	0.014	0.058	0.113	0.530	0.621
d18:2/24:0	0.135	0.595	0.662	-0.042	0.874	0.818
d18:2/16:1	ND	ND	ND	0.394	0.006	**0.025***
d18:2/18:1	-0.032	0.926	0.818	0.427	0.002	**0.012***
d18:2/20:1	NA	NA	NA	0.472	0.001	**0.004***
d18:2/22:1	NA	NA	NA	0.441	0.001	**0.009***
d18:2/24:1	0.078	0.610	0.662	0.288	0.040	0.103

Pearson correlation analysis was used to determine associations between CSF and serum ceramide and sphingomyelin lipids (natural log-transformed). [1]Correlation of coefficients (r), [2]unadjusted and [3]adjusted p-values for multiple comparisons using the two-stage Benjamini, Krieger and Yekutieli FDR method (Q-value) are reported. Correlations significant ($Q < 0.05$) are marked with an asterisk. List of abbreviations: NA, sphingolipid was not assessed; ND, sphingolipid was not detected.

Supplementary Table 3. List of ceramide and sphingomyelin standards used as either internal or quality controls. *All standards were purchased from Avanti, through their distributor Merck.

Lipid	Product ID*	pmoles on column		
		Int Std	QC (L)	QC (H)
Cer (d18:1/17:0)	860517	1000		
SM (d18:1/12:0)	860583	500		
Cer (d18:1/16:0)	860516		6.4	100
Cer (d18:0/16:0)	860634		6.4	100
Cer (d18:1/18:1)	860519		6.4	100
Cer (d18:1/18:0)	860518		6.4	100
Cer (d18:0/18:0)	860627		6.4	100
Cer (d18:1/20:0)	860520		6.4	100
Cer (d18:1/22:0)	860501		6.4	100
Cer (d18:1/24:1)	860525		6.4	100
Cer (d18:1/24:0)	860524		6.4	100
Cer (d18:0/24:0)	860628		6.4	100
SM (d18:1/16:1)	860684		3.2	50
SM (d18:1/16:0)	860584		3.2	50
SM (d18:1/18:1)	860587		3.2	50
SM (d18:1/18:0)	860586		3.2	50
SM (d18:1/24:1)	860593		3.2	50
SM (d18:1/24:0)	860592		3.2	50

Supplementary References

1. Couttas, T.A., N. Kain, A.K. Suchowerska, et al., *Loss of ceramide synthase 2 activity, necessary for myelin biosynthesis, precedes tau pathology in the cortical pathogenesis of Alzheimer's disease.* Neurobiol Aging, 2016. **43**: p. 89-100.
2. Couttas, T.A., N. Kain, C. Tran, et al., *Age-Dependent Changes to Sphingolipid Balance in the Human Hippocampus are Gender-Specific and May Sensitize to Neurodegeneration.* J Alzheimers Dis, 2018. **63**(2): p. 503-514.
3. Couttas, T.A., Y.H. Rustam, H. Song, et al., *A Novel Function of Sphingosine Kinase 2 in the Metabolism of Sphinga-4,14-Diene Lipids.* Metabolites, 2020. **10**(6).
4. Lei, M., J.D. Teo, H. Song, et al., *Sphingosine Kinase 2 Potentiates Amyloid Deposition but Protects against Hippocampal Volume Loss and Demyelination in a Mouse Model of Alzheimer's Disease.* J Neurosci, 2019. **39**(48): p. 9645-9659.
5. Lemay, A.M., O. Courtemanche, T.A. Couttas, et al., *High FA2H and UGT8 transcript levels predict hydroxylated hexosylceramide accumulation in lung adenocarcinoma.* J Lipid Res, 2019. **60**(10): p. 1776-1786.
6. Turner, N., X.Y. Lim, H.D. Toop, et al., *A selective inhibitor of ceramide synthase 1 reveals a novel role in fat metabolism.* Nat Commun, 2018. **9**(1): p. 3165.
7. Don, A.S., J.H. Hsiao, J.M. Bleasel, et al., *Altered lipid levels provide evidence for myelin dysfunction in multiple system atrophy.* Acta Neuropathol Commun, 2014. **2**: p. 150.
8. Aji, G., Y. Huang, M.L. Ng, et al., *Regulation of hepatic insulin signaling and glucose homeostasis by sphingosine kinase 2.* Proc Natl Acad Sci U S A, 2020. **117**(39): p. 24434-24442.
9. Vu, N., M. Narvaez-Rivas, G.Y. Chen, et al., *Accurate mass and retention time library of serum lipids for type 1 diabetes research.* Anal Bioanal Chem, 2019. **411**(23): p. 5937-5949.
10. Song, H., H.P. McEwen, T. Duncan, et al., *Sphingosine kinase 2 is essential for remyelination following cuprizone intoxication.* Glia, 2021. **69**(12): p. 2863-2881.
11. Marian, O.C., J.D. Teo, J.Y. Lee, et al., *Disrupted myelin lipid metabolism differentiates frontotemporal dementia caused by GRN and C9orf72 gene mutations.* Acta Neuropathol Commun, 2023. **11**(1): p. 52.
12. Mi, S., Y.Y. Zhao, R.F. Dielschneider, et al., *An LC/MS/MS method for the simultaneous determination of individual sphingolipid species in B cells.* J Chromatogr B Analyt Technol Biomed Life Sci, 2016. **1031**: p. 50-60.
13. Huang, H., T.T. Tong, L.F. Yau, et al., *LC-MS Based Sphingolipidomic Study on A2780 Human Ovarian Cancer Cell Line and its Taxol-resistant Strain.* Sci Rep, 2016. **6**: p. 34684.
14. Huynh, K., C.K. Barlow, K.S. Jayawardana, et al., *High-Throughput Plasma Lipidomics: Detailed Mapping of the Associations with Cardiometabolic Risk Factors.* Cell Chem Biol, 2019. **26**(1): p. 71-84 e4.
15. Basit, A., D. Piomelli, and A. Armirotti, *Rapid evaluation of 25 key sphingolipids and phosphosphingolipids in human plasma by LC-MS/MS.* Anal Bioanal Chem, 2015. **407**(17): p. 5189-98.
