# Supplementary material for: ReTimeML: a retention time predictor that supports the LC–MS/MS analysis of sphingolipids
Source: Sci Rep. 2024 Feb 22;14:4375. doi: 10.1038/s41598-024-53860-0 (PMC10883992; doi:10.1038/s41598-024-53860-0)
Supplement: Supplementary file 2 — Supplementary Legends. [file 41598_2024_53860_MOESM2_ESM.pdf]

Description of Additional Supplementary Files

File Name: Supplementary Data 1
Description: Regression model training results for ceramide and sphingomyelin RTs.

File Name: Supplementary Data 2
Description: Details of the published datasets for regression model training of RT estimations. The table includes the ceramide and sphingomyelin lipids assessed in each study, alongside details of chromatography conditions.

File Name: Supplementary Data 3
Description: Participants' individual ceramide and sphingomyelin concentrations (pmol/mL) for CSF and serum, quantified using LC-MS/MS.

File Name: Supplementary Data 4
Description: Template ceramide data file (.csv) for use with ReTimeML.

File Name: Supplementary Data 5
Description: Template sphingomyelin data file (.csv) for use with ReTimeML.
